# Implications of variable synaptic weights for rate and temporal coding of cerebellar outputs

**DOI:** 10.7554/eLife.89095

**Published:** 2024-01-19

**Authors:** Shuting Wu, Asem Wardak, Mehak M Khan, Christopher H Chen, Wade G Regehr

**Affiliations:** 1 Department of Neurobiology, Harvard Medical School Boston United States; 2 Department of Neural and Behavioral Sciences, Pennsylvania State University College of Medicine Hershey United States; Brandeis University United States; Brandeis University United States

**Keywords:** cerebellum, rate code, time code, disinhibition, Purkinje cell, deep cerebellar nuclei, Mouse

## Abstract

Purkinje cell (PC) synapses onto cerebellar nuclei (CbN) neurons allow signals from the cerebellar cortex to influence the rest of the brain. PCs are inhibitory neurons that spontaneously fire at high rates, and many PC inputs are thought to converge onto each CbN neuron to suppress its firing. It has been proposed that PCs convey information using a rate code, a synchrony and timing code, or both. The influence of PCs on CbN neuron firing was primarily examined for the combined effects of many PC inputs with comparable strengths, and the influence of individual PC inputs has not been extensively studied. Here, we find that single PC to CbN synapses are highly variable in size, and using dynamic clamp and modeling we reveal that this has important implications for PC-CbN transmission. Individual PC inputs regulate both the rate and timing of CbN firing. Large PC inputs strongly influence CbN firing rates and transiently eliminate CbN firing for several milliseconds. Remarkably, the refractory period of PCs leads to a brief elevation of CbN firing prior to suppression. Thus, individual PC-CbN synapses are suited to concurrently convey rate codes and generate precisely timed responses in CbN neurons. Either synchronous firing or synchronous pauses of PCs promote CbN neuron firing on rapid time scales for nonuniform inputs, but less effectively than for uniform inputs. This is a secondary consequence of variable input sizes elevating the baseline firing rates of CbN neurons by increasing the variability of the inhibitory conductance. These findings may generalize to other brain regions with highly variable inhibitory synapse sizes.

## Introduction

The cerebellum is involved in behaviors ranging from balance, motor control, and motor learning, to social and emotional behaviors (Hull and Regehr, 2022). Cerebellar dysfunction has been linked to severe motor impairment and psychiatric disorders, including autism spectrum disorder, schizophrenia, bipolar disorder, and depression (Argyropoulos et al., 2020; Schmahmann and Sherman, 1998). Within the cerebellar cortex, mossy fiber inputs from many brain regions excite granule cells that in turn activate Purkinje cells (PCs), which are the sole outputs. PCs project primarily to the cerebellar nuclei (CbN, also known as deep cerebellar nuclei [DCN]), which then project to other brain regions. Clarifying how PCs control the firing of CbN neurons is a vital step in understanding cerebellar processing.

PCs are GABAergic and fire spontaneously at frequencies ranging from 10 spikes/s to over 100 spikes/s (Thach, 1968; Zhou et al., 2014). It was estimated that 40 PCs converge onto each CbN glutamatergic projection neuron to strongly suppress their firing (Person and Raman, 2012a). This is offset by the tendency of CbN neurons to fire spontaneously (Raman et al., 2000) and by numerous excitatory inputs from climbing fiber (CF) and mossy fiber collaterals (Najac and Raman, 2017; Wu and Raman, 2017). Excitatory inputs to CbN neurons are small and slow, and their number and firing rates are poorly constrained (Najac and Raman, 2017; Wu and Raman, 2017). Therefore, in this study, we mainly focus on the control of CbN neuron firing by inhibitory inputs from PCs.

It has been proposed that PCs convey information with a rate code, a temporal code, or a combination of both, but there has been considerable debate regarding whether PC outputs are primarily encoded by firing rate or by a combination of rate and timing (Abbasi et al., 2017; Brown and Raman, 2018; Cao et al., 2012; Gauck and Jaeger, 2000; Heck et al., 2013; Herzfeld et al., 2023a; Hoehne et al., 2020; Hong et al., 2016; Medina and Khodakhah, 2012; Payne et al., 2019; Person and Raman, 2012a; Person and Raman, 2012b; Sedaghat-Nejad et al., 2022; Stahl et al., 2022; Sudhakar et al., 2015; Walter and Khodakhah, 2009; Wu and Raman, 2017). There is compelling evidence that a rate code is used for some behaviors where the firing rates of PCs and CbN neurons are inversely correlated, and the behavior outputs can be readily explained by the changes in the firing rate of PCs and CbN neurons (Abbasi et al., 2017; Herzfeld et al., 2023a; Hong et al., 2016; Medina and Khodakhah, 2012; Payne et al., 2019; Sudhakar et al., 2015; Walter and Khodakhah, 2009). On the other hand, synchronous firing or pauses of PCs allow precise temporal control of CbN neuron firing (De Schutter and Steuber, 2009; Gauck and Jaeger, 2000; Han et al., 2020; Hong et al., 2016; Özcan et al., 2020; Person and Raman, 2012a; Sedaghat-Nejad et al., 2022; Steuber et al., 2007). Dynamic clamp studies of CbN neurons with uniform-size PC inputs demonstrated the ability of PC synchrony to promote CbN neuron firing and precisely entrain spike timing (Gauck and Jaeger, 2000; Person and Raman, 2012a), but whether PC synchrony is prominent in vivo has been debated (Herzfeld et al., 2023a; Sedaghat-Nejad et al., 2022). Synchronous pauses of PC firing have also been shown to be highly effective at transiently elevating CbN neuron firing (Han et al., 2020). A recent study found that an electrically coupled subtype of molecular layer interneuron (MLI) synchronously fire and inhibit PCs in vivo (Lackey et al., 2023), thereby suggesting the presence and potential importance of synchronous pauses in vivo.

Here we reexamine how PCs control the firing of CbN neurons. We find that unitary PC-CbN inputs have highly variable amplitudes, and some are very large. We used dynamic clamp and simulations to explore the implications of nonuniform input sizes. We find that for a given total inhibitory conductance, nonuniform input sizes led to highly variable inhibitory conductances and high basal CbN firing rates. Inputs of all sizes regulated the rate and timing of CbN neuron firing, but larger inputs had a bigger influence. Although PC synchrony had less influence on the firing rates of CbN neurons for nonuniform-size inputs because of higher basal firing rates, synchronizing the firing of several large inputs effectively regulated the CbN neuron firing. Thus, the nonuniform distribution of PC input sizes allows the information of firing rates, timing, and synchrony to be conveyed from the cerebellar cortex to the CbN in a simultaneous, graded manner.

## Results

### PC to CbN input sizes are highly variable in juvenile mice

**Figure 1.**
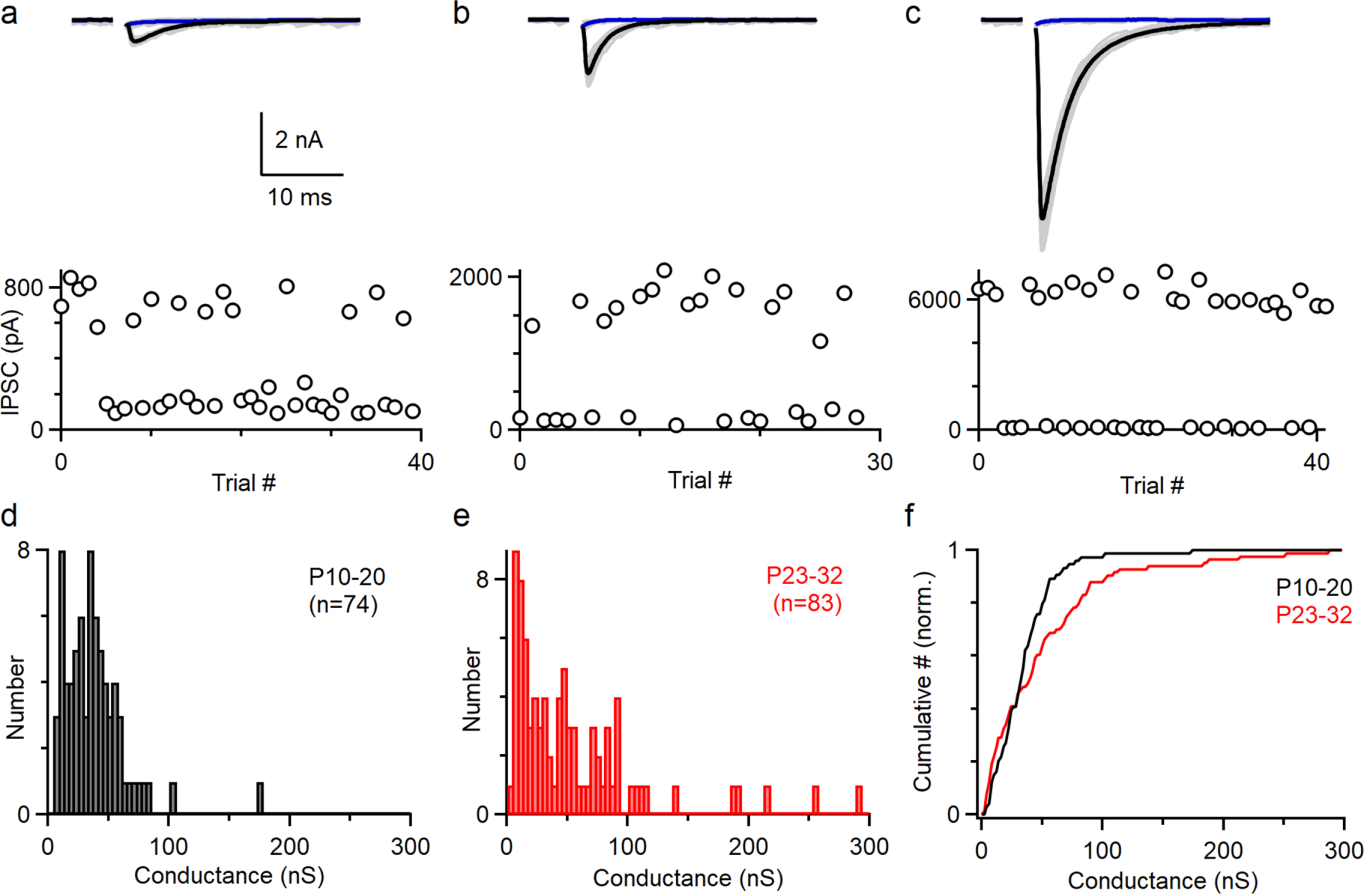
The amplitudes of Individual Purkinje cell (PC) to cerebellar nuclei (CbN) inputs are highly variable. Unitary PC-CbN IPSCs were recorded in brain slices. The data in (**a–c**) were obtained for this study. The histogram of input sizes in (**d–e**) were based on new experiments (**d**, n = 44; **e**, n = 39), on Turecek et al., 2017 (**e**, n = 44), and Khan et al., 2022 (**d**, n = 30). (**a**) Example of a small PC-CbN input (P27). Top: responses evoked with the same stimulus intensity are superimposed for 40 trials (gray), and the average of successes (black) and failures (blue) is shown. Bottom: IPSCs’ amplitudes are plotted as a function of trial number. (**b**) As in (**a**), but for a medium-size input onto the same cell. (**c**) As in (**a**), but for a large-size input onto the same cell. (**d**) Distribution of input sizes for PC-CbN IPSCs in young mice (P10–P20, n = 74). (**e**) Distribution of input sizes for PC-CbN IPSCs in juvenile mice (P10–P20, n = 83). (**f**) Normalized cumulative plot of conductances. The distributions of unitary input sizes in P10–20 animals and P23–32 animals were significantly different (p<0.0001) with a Kolmogorov–Smirnov test. Figure 1—source data 1.PC-CbN unitary IPSCs and conductances.

To understand how PCs control the firing of CbN neurons, it is necessary to determine the distribution of the sizes of PC inputs. Previously, PC to CbN inputs were approximated by uniform inputs based on the average of all inputs recorded (Han et al., 2020; Person and Raman, 2012a; Wu and Raman, 2017). We reexamined the issue of input sizes by determining the sizes of many individual inputs in young (P10-20, n = 74) and juvenile (P23-32, n = 83) animals (Figure 1). We cut brain slices, stimulated PC axons with an extracellular electrode placed far from the CbN, and recorded the evoked inhibitory postsynaptic currents (IPSCs) in CbN glutamatergic projection neurons with whole-cell recordings. We used a high Cl^-^ internal solution to determine the distribution of input sizes because it provided superior stability, low-access resistance, and high sensitivity compared to a low Cl^-^ internal. We adjusted the stimulus intensity to stochastically activate an individual PC axon and isolate a unitary input. This approach is shown for three inputs onto the same CbN neuron for a juvenile mouse (P27) (Figure 1a–c). As shown in the individual trials for a small (weak) input, stimulation evoked short-latency IPSCs (720 pA) in some trials but not others (Figure 1a, upper, gray). The average of successes (Figure 1a, upper, black) and failures (Figure 1a, upper, blue) is shown. This is also apparent in a plot of the IPSC amplitudes for each trial (Figure 1a, lower) showing that the IPSCs were evoked in a fraction of the trials. Similar experiments are shown for a medium-size input (Figure 1b, 1690 pA) and a large-size (strong) input (Figure 1c, 6280 pA). To better evaluate the variability in the input sizes, we plotted the distributions of unitary PC to CbN input conductances for young (Figure 1d, P10–20) and juvenile (Figure 1e, P23–32) mice. Input sizes were somewhat variable in young mice (coefficient of variation [CV] = 0.7; Figure 1d), which is qualitatively similar to previous findings (P13–29, n = 30) (Person and Raman, 2012a). In juvenile mice, there was considerably more variability, primarily because of the presence of many medium and large inputs (mean 52.9 ± 6 nS, CV = 1.0; Figure 1e). This is evident in a comparison of the cumulative histograms of IPSC conductances for young and juvenile animals, which showed significantly different distributions (Figure 1f; p<0.0001, Kolmogorov–Smirnov test). These results show that PC to CbN input strength is highly variable in juvenile mice.

### Using dynamic clamp and simulations to examine the effects of variable input sizes

The variability in the amplitudes of PC to CbN inputs in juvenile animals raised the issue of how the wide range of input sizes influences the way PCs control the firing of CbN neurons. We then designed dynamic clamp studies using highly variable PC-CbN input sizes. The PC-CbN unitary conductances we used in dynamic clamp studies were based on our measured input sizes with two corrections (‘Methods’). First, we corrected for the effects of a high-chloride internal solution by scaling down the amplitudes by a factor of 2.3, based on the estimates of conductance values determined with high and physiological Cl^-^ internal solutions (Bormann et al., 1987; Gjoni et al., 2018; Sakmann et al., 1983). Second, we corrected the input sizes for the depression (reduced to 40% of initial amplitude) that occurs during physiological activation (Pedroarena and Schwarz, 2003; Telgkamp and Raman, 2002; Turecek et al., 2017; Turecek et al., 2016), as has been done in previous dynamic clamp studies (Han et al., 2020; Person and Raman, 2012b; Wu and Raman, 2017). The excitatory conductances we used in dynamic clamp studies were based on previous studies (Najac and Raman, 2017; Wu and Raman, 2017), with a relatively unconstrained frequency to pair with different inhibitory conductances.

**Figure 2.**
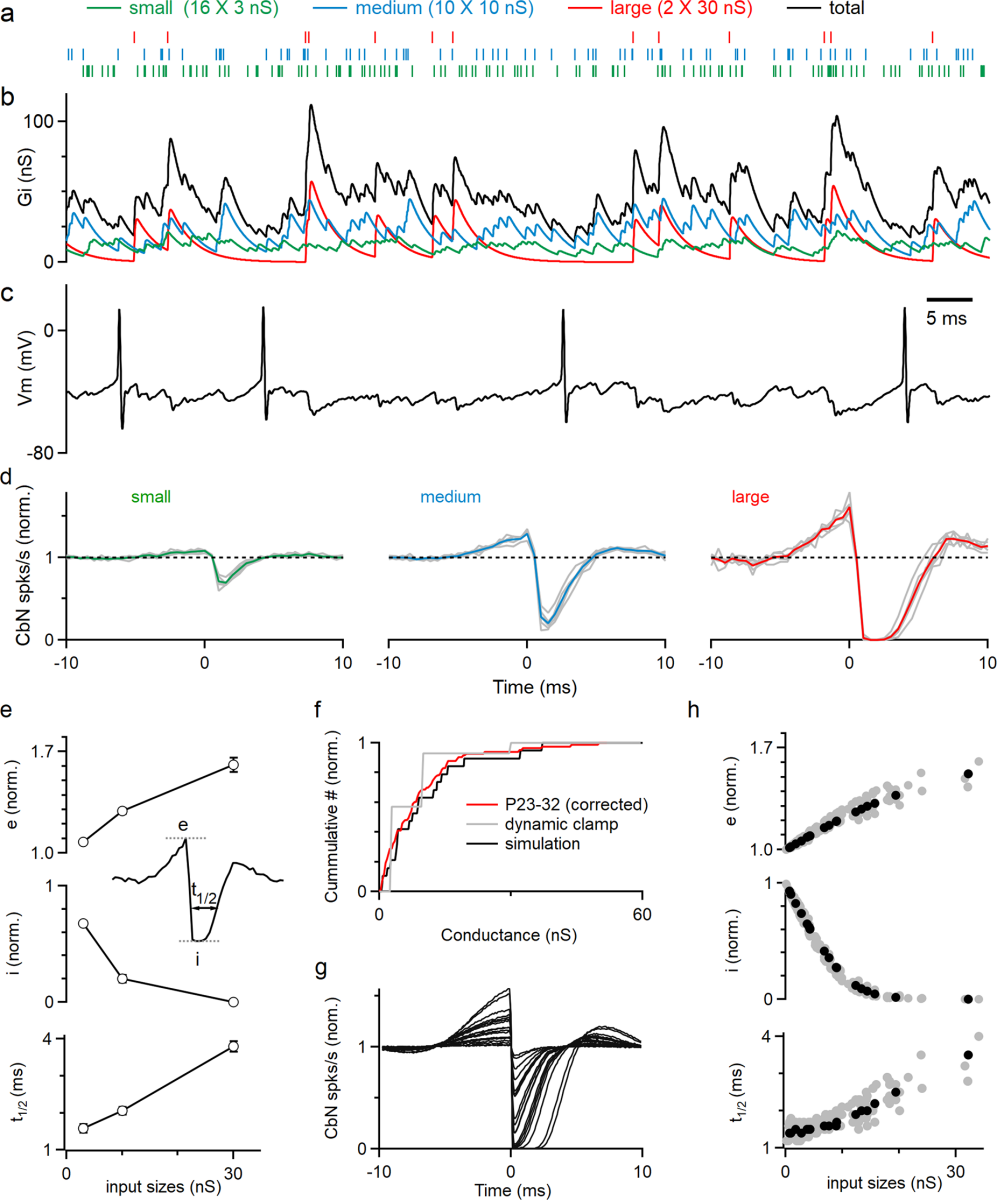
Large Purkinje cell (PC) inputs powerfully influence cerebellar nuclei (CbN) neuron firing. Dynamic clamp experiments and simulations were conducted with variable distribution of PC input sizes. (**a**) The corrected distributions of PC-CbN input sizes in juvenile animals (**f**, red) were approximated with 16 small inputs (3 nS, green), 10 medium inputs (10 nS, blue), and 2 large inputs (30 nS, red). Raster plots are shown for the spike times of the 16 small (green), 10 medium (blue), and 2 large (red) inputs used in dynamic clamp experiments. (**b**) The total conductance waveform is shown (black) along with contributions from small (green), medium (blue), and large (red) inputs. (**c**) Spikes in a CbN neuron evoked by the total conductance in (**b**) in dynamic clamp experiments. (**d**) The normalized cross-correlograms of input spiking and CbN neuron spiking for small, medium, and large inputs for dynamic clamp experiments. Different cells (n = 6, gray) are shown along with the average cross-correlograms (colored traces). (**e**) Summary of the excitation (**e**), inhibition (**i**), and half-decay time (*t*_1/2_), as defined in the inset for the data in (**d**). Inset shows how the parameters are determined. (**f**) Cumulative histogram of all recorded inputs from Figure 1 for P23–32 mice corrected for depression and internal solution (red), the simplified input distribution used in dynamic clamp studies in a (gray), and inputs drawn from that distribution that were used in a simulation (black). (**g**) Calculated cross-correlograms (normalized) for each of the different inputs used in a simulation. (**h**) Summary of the excitation (**e**), inhibition (**i**), and half-decay time (*t*_1/2_), for a simulation as defined in the inset for the simulated cell in (**g**) (black), and for inputs to nine other simulated cells with different distributions of inputs (gray). The summary data are shown as the mean ± SEM. Figure 2—source data 1.Cross-correlograms and input sizes.

The corrected distribution of input sizes used to guide our dynamic clamp studies is shown in Figure 2f (red). For simplicity, instead of having a continuous range of input sizes, we approximated the distribution of PC input sizes in juvenile animals (Figure 1e, ‘Methods’) with 16 small (3 nS), 10 medium (10 nS), and 2 large (30 nS) inputs (Figure 2f, gray; total 200 nS, similar to the total conductances used in Person and Raman, 2012a). The timing and frequency of the inputs were based on PC firing recorded in awake-behaving mice, with each input firing at 83 spikes/s (Figure 2—figure supplement 1, ‘Methods’). The resulting spike times (Figure 2a, Figure 2—figure supplement 2) were convolved with the corresponding unitary conductance size (Figure 2—figure supplement 2) to generate the small (Figure 2b, green), medium (Figure 2b, blue), and large (Figure 2b, red) conductances, which were then summed to generate the total inhibitory conductance arising from all inputs (Figure 2b, black). The total inhibitory conductance showed large variations that were dominated by the medium and large inputs (Figure 2b), which in turn produced large fluctuations in the membrane potential when injected into a CbN neuron (Figure 2c). Spiking of the CbN neuron occurred mainly when the amplitude of the total inhibitory conductance was small (Figure 2b and c).

**Figure 2—figure supplement 1.**
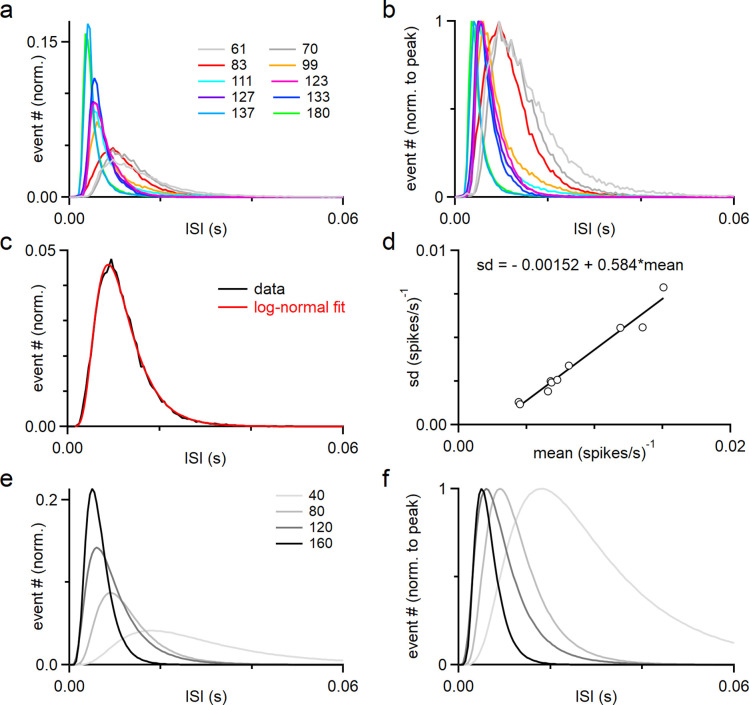
Interspike interval (ISI) histograms of Purkinje cells (PCs) firing used in this study. (**a**) Normalized ISI histograms of 10 PCs recorded in vivo from our previous studies (Han et al., 2020), with their average firing rates indicated in the legend. (**b**) As in (**a**) but normalized to the peak. (**c**) Example of a lognormal distribution fitting (red) to the ISI histogram of one PC recorded in vivo (black, 83 Hz). Similar fittings were performed for the other nine PCs. (**d**) The standard deviation (sd) as a function of the mean of the lognormal distribution fits to the 10 PCs was fitted as a linear function. Fits were performed in IGOR Pro to the function.$exp\left\{{-\left[ln\left(x/x_{0}\right)/width\right]}^{2}\right\}$. The values of μ and σ for standard lognormal distribution are computed from $x_{0}$ and width using equations: μ = ,$ln\left(x_{0}\right)+{width}^{2}/2$ σ = $width/\sqrt{2}$ The values of mean and sd of the lognormal distribution fits were computed from μ and σ using equations: mean = $exp\left(\mu +\sigma^{2}/2\right)$ and sd = $\sqrt{exp\left(2\mu +\sigma^{2}\right)\left[exp\left(\sigma^{2}\right)-1\right]}$. This linear function was used to determine the parameters for a PC firing ISI lognormal distribution with a desired firing rate. (**e**) Four lognormal distributions representing artificial PC firing ISI distributions with different firing rates generated with the mean and sd from the linear function in (**d**). (**f**) As in (**e**) but normalized to the peak.

**Figure 2—figure supplement 2.**
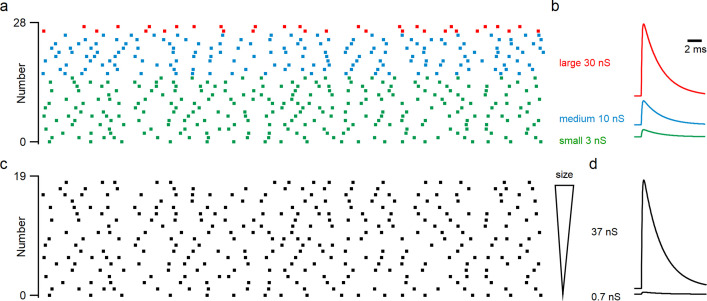
Further explanations of the dynamic clamp experiments and simulations. (**a**) Related to Figure 2a. Raster plots of 16 small inputs (green), 10 medium inputs (blue), and 2 large inputs (red) that fire at 83 Hz. (**b**) Related to Figure 2b. Unitary Purkinje cell–cerebellar nuclei (PC-CbN) input waveforms with the indicated sizes. The spikes from each different-size inputs in (**a**) were convolved with the respective waveform to obtain the total inhibitory conductance shown in Figure 2b. (**c**) Similar as (**a**) but for a simulation with 19 inputs, with their size distributed indicated in Figure 2f. (**d**) Unitary PC-CbN input waveforms with the largest size and smallest size from the simulation. The raster plot of each PC input was convolved with the appropriate size conductance waveform to obtain its contribution to the inhibitory conductance, and the total inhibitory conductance was computed by summing the conductances of all inputs.

**Figure 2—figure supplement 3.**
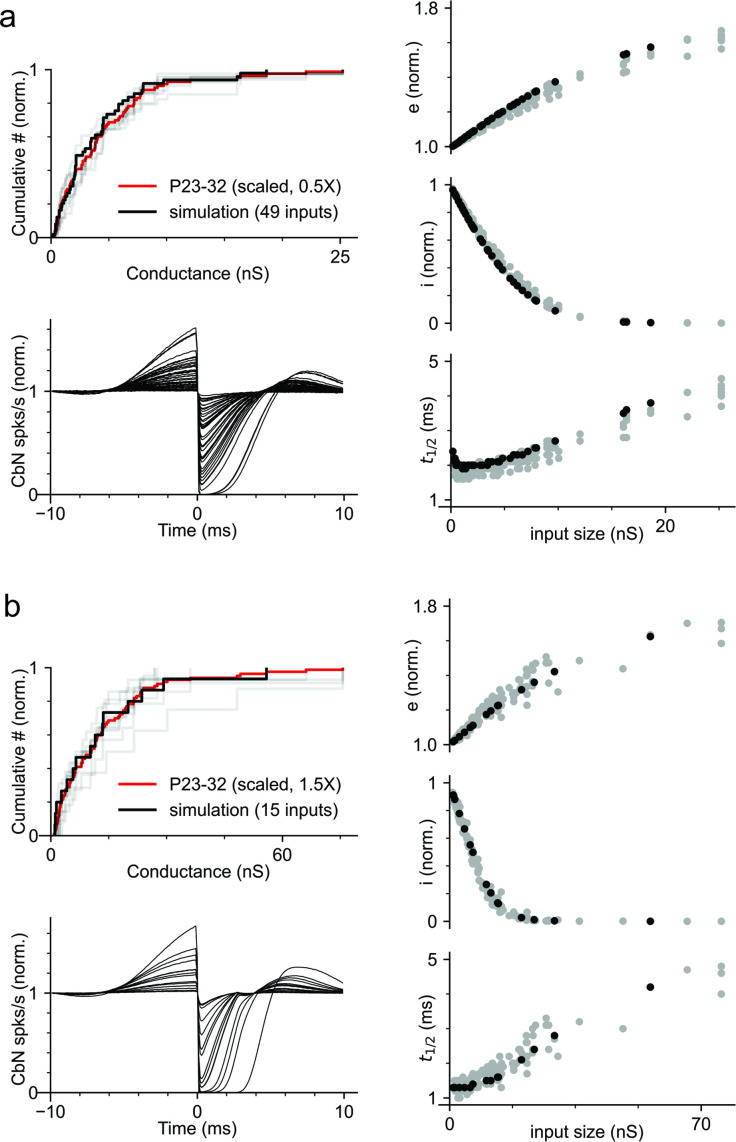
Extra simulations with different scaling factors of the unitary Purkinje cell–cerebellar nuclei (PC-CbN) conductances. (**a**) Similar to the simulation in Figure 2f–h, but with a scaling factor of 0.5. (**b**) Similar to the simulation in Figure 2f–h, but with a scaling factor of 1.5.

All inputs had effects on the timing of CbN neuron firing, but larger inputs had much bigger effects. As shown in the cross-correlograms of input timing and CbN neuron spiking, small inputs produced a small transient decrease in CbN neuron spiking (32% decrease; Figure 2d, left), while medium-size inputs strongly reduced CbN neuron spiking (80% decrease; Figure 2d, middle), and large inputs transiently shut down CbN neurons for approximately 2 ms (Figure 2d, right). Intriguingly, the inhibition generated by inhibitory inputs was preceded by an increase in spikes that was particularly prominent for large inputs (61% increase for large, 29% increase for medium, and 8% increase for small; Figure 2d). The magnitude of excitation (Figure 2e, top) and inhibition (Figure 2e, middle), as well as the duration of inhibition (Figure 2e, bottom), were all positively correlated with the size of the PC input. These findings highlight the ability of large PC inputs to control the timing of CbN neuron spiking.

We then performed simulations to extend these findings to a more realistic distribution of PC input sizes (‘Methods’). We determined the sizes of PC input by assigning them values randomly drawn from the distribution of Figure 2f (red) until the total inhibitory conductance reached 200 nS. An example distribution of PC input sizes used in a simulation is shown in Figure 2f. The cross-correlograms of input timing and CbN neuron spiking for each of the PC inputs were determined, and they all showed a similar pattern, with an elevation followed by a suppression of CbN firing (Figure 2g). The magnitudes of the excitation and inhibition of CbN firing had a similar dependence on the size of the PC inputs as shown for the example neuron (Figure 2h, black) and for nine other simulated neurons (Figure 2h, gray). In light of the many types of voltage-gated ion channels present in CbN neurons (Afshari et al., 2004; Aizenman and Linden, 1999; Alviña and Khodakhah, 2008; Raman et al., 2000; Raman and Bean, 1999; Santoro et al., 2000; Steuber et al., 2011), we were somewhat surprised that the simple integrate-and-fire model we used was able to recapitulate our dynamic clamp studies, which indicates that our conclusions are not specific to the properties of the CbN neurons. We also performed simulations with different scaling factors of the distribution of input sizes, while keeping the same total conductances (Figure 2—figure supplement 3). Applying a scaling factor of 0.5 or 1.5 changed the number and size of PC inputs, but the results were qualitatively similar, and in all cases larger inputs had a greater influence on the firing of CbN neurons. These simulations complemented our dynamic clamp studies and showed that the properties of cross-correlograms and their dependence on the size of the PC input can be extended to realistic distributions of input sizes.

### The autocorrelation of PC firing leads to disinhibition prior to inhibition of CbN neurons

**Figure 3.**
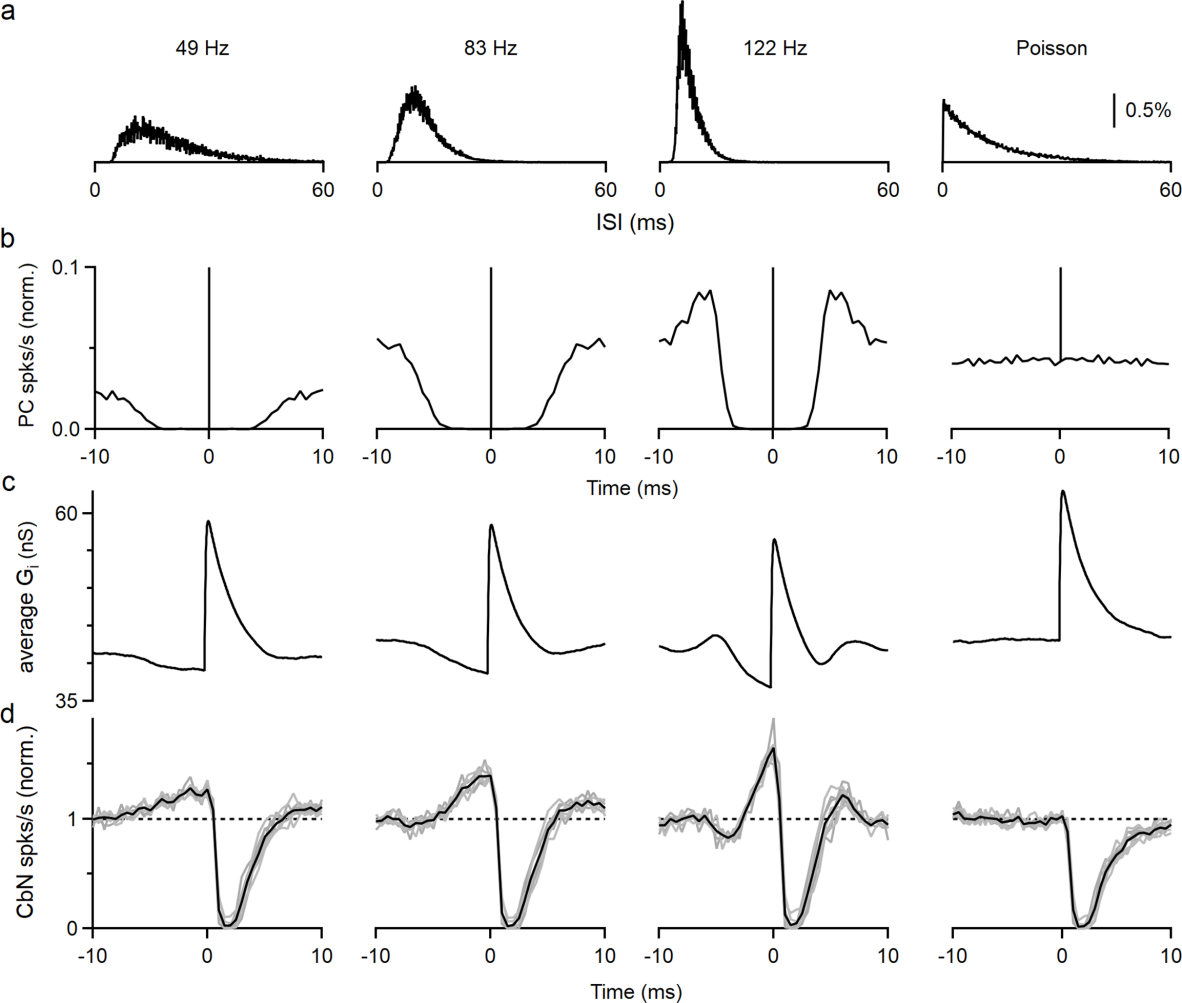
Autocorrelation of Purkinje cell (PC) firing leads to excitation prior to inhibition of cerebellar nuclei (CbN) neurons. Dynamic clamp experiments were conducted using PCs with different firing statistics. (**a**) Interspike interval (ISI) histograms for three different PCs recorded in vivo (left), and for an artificial Poisson input lacking a refractory period (right). (**b**) Autocorrelation functions for the ISI distributions in (**a**). At 0 ms, all graphs peak at 1 and graphs are truncated to allow better visualization. (**c**) Calculated spike-triggered average inhibitory conductances for different cases (12 × 20 nS at 49 Hz, 9 × 20 nS at 83 Hz, 6 × 20 nS at 122 Hz, and 9 × 20 nS for Poisson inputs), with ISIs drawn from the corresponding distributions in (**a**). (**d**) Cross-correlograms are shown for PC inputs and CbN spiking for dynamic clamp experiments that used the distributions in (**a**).

The elevated spiking observed prior to the inhibition by a PC input is intriguing. If such a cross-correlogram was observed in vivo, it might be attributed to excitation of the CbN neuron by other inputs that preceded PC inhibition, but that cannot be the case for our experimental conditions. Another possibility is that the refractory period of PC firing results in reduced inhibition and effective excitation. In that case, the properties of this disinhibition period will be shaped by the firing statistics of PCs. We tested this hypothesis by performing dynamic clamp experiments with the timing based on three different PCs recorded in vivo and on an artificial Poisson distribution (Figure 3a). As shown in the interspike interval (ISI) histograms (Figure 3a), auto-correlograms (Figure 3b), and in raster plots (Figure 3—figure supplement 1a), the three recorded PCs had refractory periods that were correlated with their firing frequency (~3.5 ms for 49 Hz, ~2 ms for 83 Hz, and ~1.5 ms for 122 Hz firing), while the Poisson input did not have a refractory period. We then generated inhibitory conductances by convolving spike times with a 20 nS single-input conductance. To compensate for the differences in input firing frequency, we adjusted the number of the inputs so that the total inhibitory conductances were approximately the same for all cases (12 × 20 nS at 49 Hz, 9 × 20 nS at 83 Hz, 6 × 20 nS at 122 Hz, and 9 × 20 nS for Poisson inputs). When input spike timing was based on in vivo recordings of PC firing, the average inhibitory conductances decreased prior to the PC spike as a result of the refractory period (Figure 3c). Consequently, the cross-correlograms (Figure 3d) and raster plots (Figure 3—figure supplement 1b) showed elevated spiking in the CbN neuron prior to suppression by the PC spike. This is because a PC that spikes at *t* = 0 does not spike for several milliseconds before or after that spike. Consistent with our hypothesis, the speed and amplitude of this decrease in inhibitory conductance and the associated elevation in spiking depended on the PC firing statistics, with the faster firing inputs associated with larger, shorter-lived effects (49 Hz: 27% increase, halfwidth 5.3 ms; 83 Hz: 39% increase, halfwidth 3.4 ms; 122 Hz: 65% increase, halfwidth 1.7 ms; Figure 3d). The cross-correlograms of PC inputs and CbN firing transiently showed transient suppression of firing that was followed by briefly elevated firing that was most prominent for rapidly firing PCs (Figure 3d, PCs that fire at 122 spikes/s). This elevated firing was also readily explained by the properties of PC firing and the refractory period of PCs. The Poisson input that lacked a refractory period did not have a decrease in the inhibitory conductance, and therefore CbN firing was not elevated prior to or following inhibition in the cross-correlogram (Figure 3c and d, far right). Thus, the autocorrelations of PC firing led to different extents of elevated CbN firing prior to suppression, and this affects how they control the spike timing of CbN neurons.

**Figure 3—figure supplement 1.**
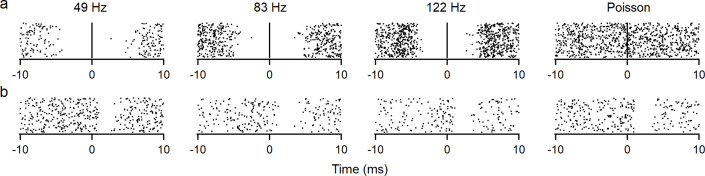
Raster plots of the autocorrelations and cross-correlations. (**a**) Relevant to Figure 3b. Raster plots showing 500 sweeps of autocorrelations of Purkinje cells (PCs) firing at different frequencies and PCs firing in a Poisson fashion. The refractoriness of PC firing reflects their firing statistics. (**b**) Relevant to Figure 3d. Raster plots showing 500 sweeps of the spike trains of a cerebellar nuclei (CbN) neuron surrounding same-sized PC inputs firing at different frequencies and PCs firing in a Poisson fashion. The elevation of CbN neuron firing prior to the timing of the PC input is most apparent for rapidly firing inputs and is absent from the Poisson input that lacks a refractory period.

### The amplitude and coefficient of variation of PC inhibition regulate the firing rate of CbN neurons

**Figure 4.**
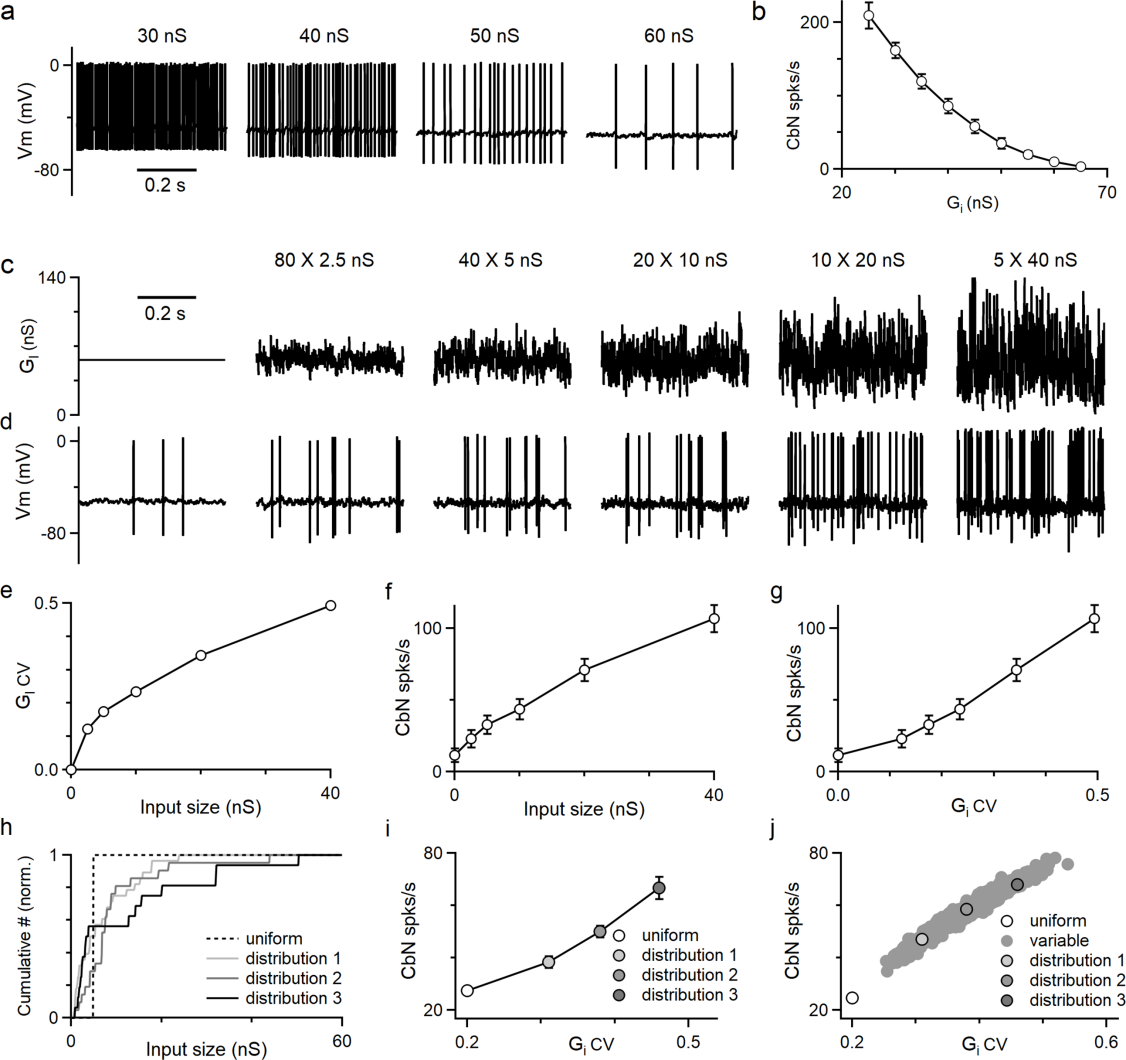
The amplitudes and fluctuations of the total inhibitory conductance both regulate the firing of cerebellar nuclei (CbN) neurons. Dynamic clamp experiments and simulations were conducted with varied amplitudes and fluctuations in the total inhibitory conductance. (**a**) CbN neuron spiking observed for constant inhibitory conductances of the indicated amplitudes. (**b**) Summary of CbN neuron firing rates (n = 7 cells) during constant inhibitory conductances as in (**a**). (**c**) Inhibitory conductances with the same average conductance (56 nS) are shown for a constant conductance (far left) and for different cases with the numbers and sizes of inputs varied. (**d**) CbN neuron spiking is shown for dynamic clamp experiments with the inhibitory conductances in (**c**). (**e**) The coefficient of variation (CV) of the inhibitory conductance is plotted as a function of the input size. (**f**) CbN firing rate is plotted as a function of input size. (**g**) CbN firing rate is plotted as a function of the CV of the inhibitory conductance. (**h**) Normalized cumulative plots of conductances of three different input distributions (solid lines) drawn from the observed distribution (Figure 2f, red) and for the 40 × 5 nS inputs (dashed line). (**I**) CbN firing rates in dynamic clamp experiments that used the inhibitory conductances in (**h**) are plotted as a function of the CV of the inhibitory conductance. (**j**) Simulated CbN firing rates based on 1000 different input distributions randomly drawn from the observed distribution of input sizes (filled gray) and for 40 × 5 nS inputs (open circle) are plotted as a function of the CV of the inhibitory conductance. The three distributions used in dynamic clamp experiments in (**i**) are highlighted. The summary data are shown as the mean ± SEM. Figure 4—source data 1.Different distributions and CbN neuron firing rates.

Thus far, we have shown the differential effects on CbN spike timing by different-size PC inputs. PCs also control the average firing rate of CbN neurons in addition to spike timing. We used dynamic clamp to determine how the amplitude and the CV of the inhibitory conductance influence CbN firing. We began by examining how changing the amplitude of a constant inhibitory conductance altered the firing of CbN neurons (Figure 4a). The firing rate of CbN neurons was approximately 200 spikes/s for an inhibitory conductance of 30 nS, but as the magnitude of the inhibitory conductance increased the spike rate decreased, and an inhibitory conductance of 65 nS silenced CbN firing (Figure 4a and b). As expected, the CbN firing rate was inversely related to the amplitude of inhibitory conductance (Figure 4b). We then examined how the variability in the inhibitory conductance influenced CbN spiking. We kept the total inhibitory conductance constant while varying the sizes and numbers of PC inputs that contribute to the total inhibitory conductance (Figure 4c). We generated the total inhibitory conductance for the indicated numbers and sizes of PC synaptic inputs with the timing based on PC firing recorded in vivo (Figure 4c, Figure 2—figure supplement 1; ‘Methods’). While the average of the conductance wave was the same for all combinations of input sizes and numbers, as the size of the inputs increased, the conductance became increasingly variable (Figure 4c) and the CV became larger (Figure 4e). Evoked firing rates in CbN neurons were low for many small inputs, but as the number of inputs decreased and the size increased, the firing rate increased markedly (from 12 spikes/s to 107 spikes/s; Figure 4d and f). The firing rate was strongly dependent on the CV of the inhibitory conductance (Figure 4g). These findings establish that increases in the magnitude of the average inhibitory conductance suppress firing, whereas increases in the variability of the inhibitory conductance promote firing.

The dependence of CbN firing on the variability of the inhibitory conductance prompted us to examine the influence of variable input sizes on the basal firing rate of CbN neurons. Three different distributions of PC input sizes drawn from the observed distribution of input sizes (Figure 2f, red) with different CVs and a total conductance value of 200 nS (Figure 4h, solid lines), together with 40 uniform 5 nS PC inputs (Figure 4h, dashed line), were used to generate inhibitory conductances for dynamic clamp experiments. Even though the average conductance was the same, the firing rates for the three nonuniform input sizes were higher than for uniform inputs (38, 50, and 67 spikes/s vs. 27 spikes/s; Figure 4i). The differences in the firing rates are readily explained by the CV of total conductances generated from different-size inputs (Figure 4i). We also simulated the firing evoked by 1000 different distributions of PC input sizes drawn from the observed input size distribution with a total conductance size of 200 nS and observed a broad range of firing rates that depended on the CV of the total inhibitory conductance (Figure 4j). Both dynamic clamp experiments and simulations showed that for the same total inhibitory conductance, CbN neuron firing rates are always higher for nonuniform inputs than for uniform inputs as a result of the higher CV of the inhibitory conductance (Figure 4i and j).

### Different-size inputs reliably transfer a rate code

**Figure 5.**
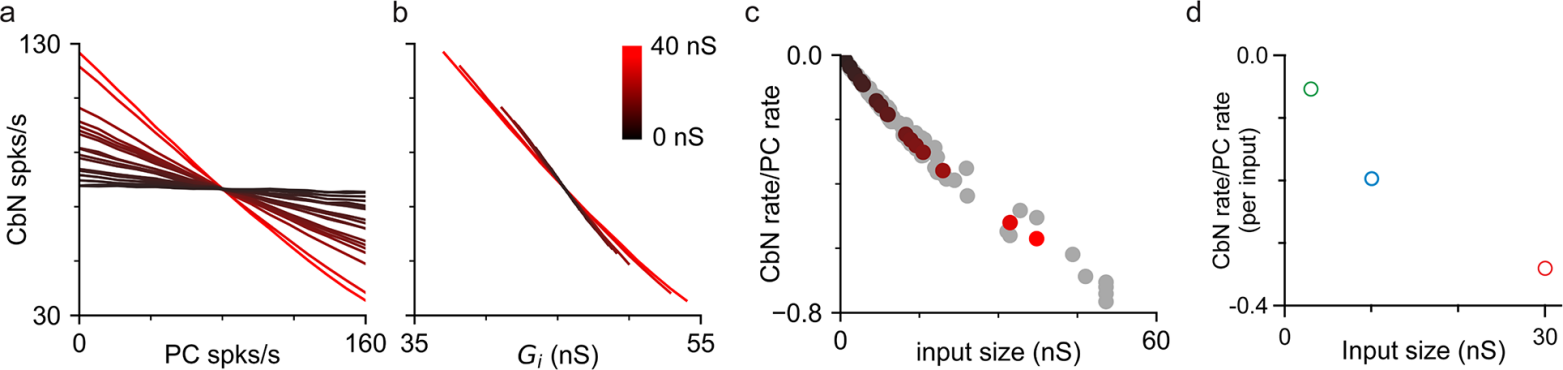
Purkinje cell (PC) inputs effectively convey an inverse rate code. Simulations and dynamic clamp experiments were conducted with the firing rates of PC inputs varied. (**a**) Simulations with different-size inputs drawn from the distribution in Figure 2f and the firing rates of each input were varied. The firing rates of cerebellar nuclei (CbN) neurons are plotted as a function of the firing rate of the varied PC input, with the color indicating the input size. (**b**) The firing rates of CbN neurons in (**a**) are plotted as a function of the total inhibitory conductance (*G*_i_) resulting from varying the firing rate of each PC input. The color scale is the same as in (**a**). (**c**) The slopes of CbN output firing rates vs. PC input firing rates are plotted for different-size inputs for the simulated CbN neuron in (**a**) and (**b**) (black), and nine other neurons with different input size distributions (gray). (**d**) Dynamic clamp experiments were performed with small (16 × 3 nS, green), medium (10 × 10 nS, blue), and large (2 × 30 nS, red) inputs. The firing rates of either all small, all medium, or all large inputs were varied (Figure 5—figure supplement 2). The slope of CbN output firing rate vs. PC input firing rate divided by the number of inputs is plotted for different-size inputs. Figure 5—source data 1.Simulations and dynamic clamp experiments for rate codes.

The ability of different-size PC inputs to convey a rate code to CbN neurons was unclear, given that both the magnitude and variability of total inhibition can influence the firing rates of CbN neurons. We first addressed this issue using simulations, in which we varied the firing rates of each PC input individually to determine how faithfully they convey a rate code. Simulations were similar to those of Figure 2g and h, but with the firing rates for each input varied between 0 and 160 spikes/s while maintaining the average firing rates of other inputs at 80 Hz (‘Methods’). Varying the firing rate of individual PC inputs led to an approximately linear, negatively correlated change in CbN firing rate (Figure 5a). Single large inputs were surprisingly effective at regulating the firing rates of CbN neurons. In this example, varying a single 37 nS input from 0 to 160 spikes/s decreased the output firing frequency from 126 spikes/s to 35 spikes/s (Figure 5a, red). There was a remarkably consistent relationship between the firing rates of CbN neurons and the total inhibitory conductances (Figure 5b). The slope of the CbN output firing rate/PC input firing rate was negatively correlated with the input size for the example neuron (Figure 5c, color coded) and for nine other neurons (Figure 5c, gray), and when different scaling factors were used (Figure 5—figure supplement 1). We also address this issue using dynamic clamp experiments in which the frequencies of small, medium, or large inputs were varied (Figure 5—figure supplement 2) and found that the ability of individual inputs to suppress CbN neuron firing also depended on input size (Figure 5d). These studies establish that individual PC inputs convey a rate code that depends on input size.

**Figure 5—figure supplement 1.**
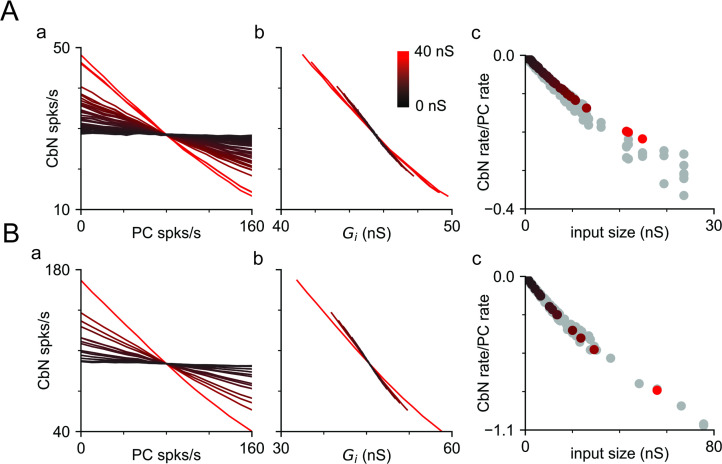
Simulations with different scaling factors of the unitary Purkinje cell–cerebellar nuclei (PC-CbN) conductances. (**A**) Similar to the simulation in Figure 5a–c, but with a scaling factor of 0.5 for the input sizes. (**a**) Simulations with different-size inputs drawn from the distribution in Figure 2f and the firing rates of each input were varied. (**b**) The firing rates of CbN neurons in (**a**) are plotted as a function of the total inhibitory conductance (*G*_i_) resulting from varying the firing rate of each PC input. (**c**) The slope of CbN output firing rate vs. PC input firing rate is plotted for different inputs for the simulated CbN neuron in (**a**) and (**b**) (black), and for nine other neurons with different input distributions (gray). (**B**) As in (**A**), but with a scaling factor of 1.5 for the input sizes.

**Figure 5—figure supplement 2.**
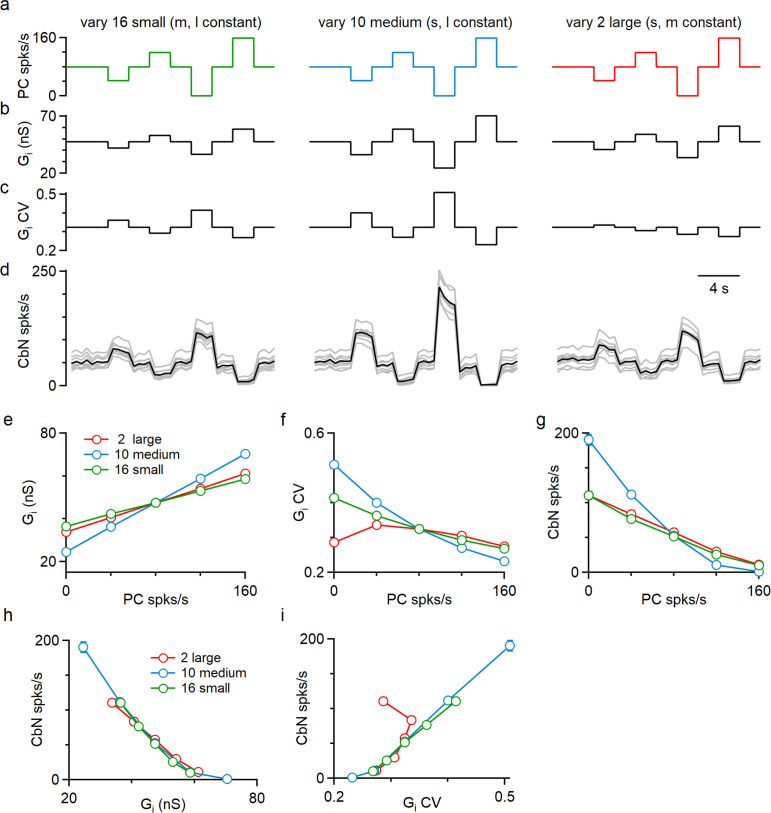
Dynamic clamp experiments with small (16 × 3 nS, green), medium (10 × 10 nS, blue), and large (2 × 30 nS, red) inputs. The firing rates of either small, medium, or large Purkinje cell (PC) inputs were varied from 0 to 160 spikes/s. (**a**) The firing rates of either small (green), medium (blue), or large (red) inputs were varied every 2 s while the firing rates of the other inputs were maintained at 80 spikes/s. (**b**) The resulting average inhibitory conductances for the three different conditions are shown. (**c**) The resulting average coefficient of variation (CV) of the inhibitory conductances is shown. (**d**) The firing rates of cerebellar nuclei (CbN) neurons with the conductances in (**a–c**) are shown as individual cells (n = 9, gray) and their average (black). (**e**) Quantification plot for (**b**). Total inhibitory conductance is plotted as a function of the firing rate of the varied PC inputs. Elevating the firing rates of all size inputs increased the amplitude of the total inhibitory conductance. Varying the firing rate of all medium inputs had larger effects because of their larger contribution to the inhibitory conductance. (**f**) Quantification plot for (**c**). The CV of the total inhibitory conductance is plotted as a function of the firing rate of the varied PC inputs. Eliminating the firing of either the small or medium inputs increased the CV of the inhibitory conductance, whereas eliminating the firing of the large inputs decreased the CV of the inhibitory conductance. (**g**) Quantification plot for (**d**). The firing rates of CbN neurons are plotted as a function of the firing rate of the varied PC inputs. (**h**) The firing rates of CbN neurons are plotted as a function of the total inhibitory conductance (*G*_i_). (**I**) The firing rates of CbN neurons are plotted as a function of the CV of total inhibitory conductance (*G*_i_ CV). The summary data are shown as the mean ± SEM.

### Synchronous firing of uniform- or variable-size PC inputs

**Figure 6.**
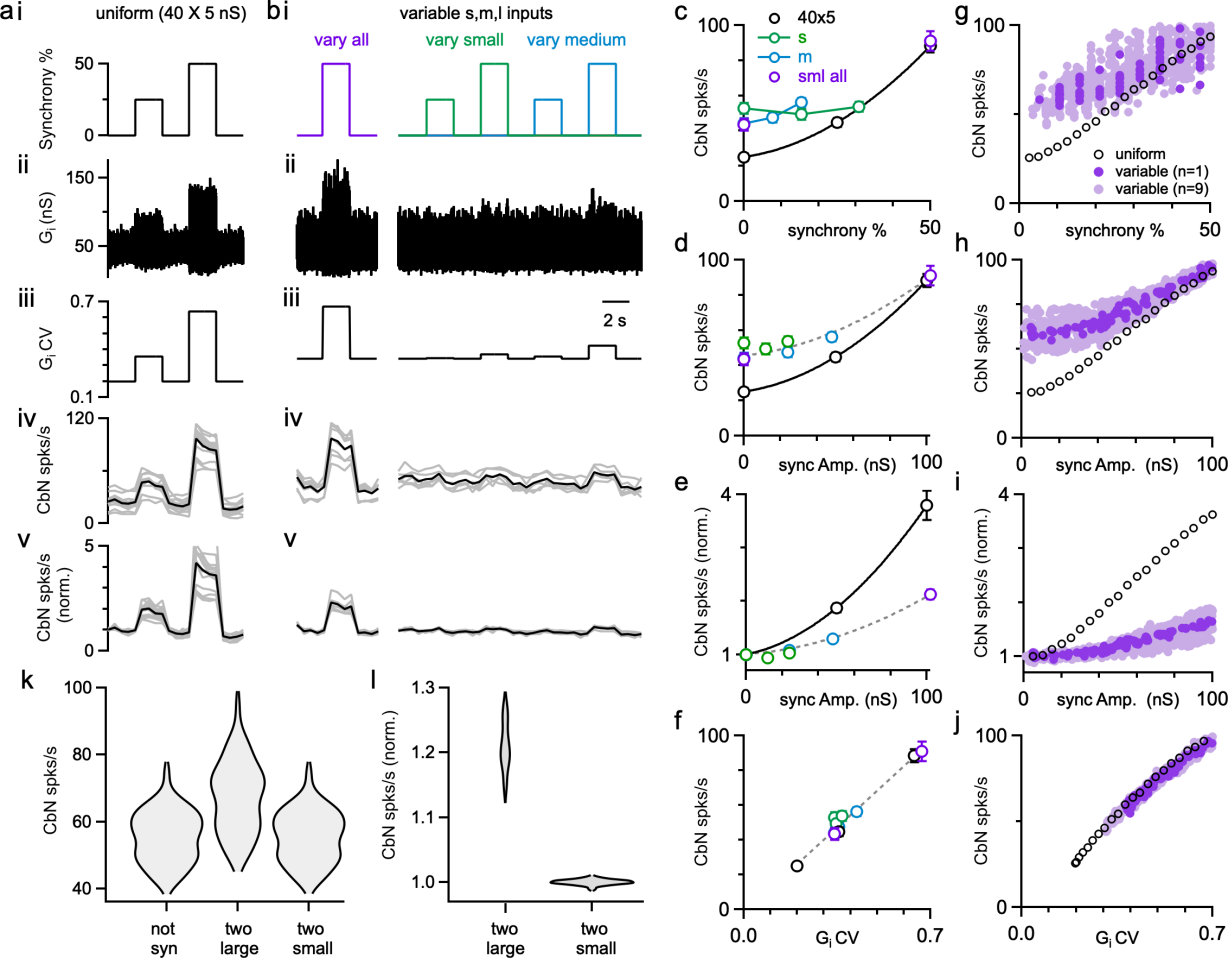
The influence of Purkinje cell (PC) synchrony on the firing rates of cerebellar nuclei (CbN) neurons for uniform- and variable-size PC inputs. Dynamic clamp experiments were conducted with either uniform (**a**) or variable inputs (**b**) in which the conductance was kept constant, but the synchrony of the inputs was varied. (**a**) (**i**) The extent of synchrony of uniform-size inputs (40 × 5 nS) is varied. (**ii**) The inhibitory conductance in which the synchrony of inputs is varied as in (**a**). (**iii**) The coefficient of variation (CV) of the inhibitory conductance. (**iv**) CbN neuron firing rate with the conductance (average, black; individual cells, n = 14, gray). (**v**) The normalized firing rate for the cells in (**iv**). (**b**) Similar experiments were performed on the same cells as in (**a**), but for inputs of variable-sizes (small: 16 × 3 nS, green; medium: 12 × 8 nS, blue; large: 2 × 30 nS, red). In some cases, all types of inputs were synchronized (purple), in others only the small inputs (green) or the medium inputs (blue) were synchronized. (**c**) CbN neuron firing rate as a function of the percentage of synchronous inputs. (**d**) CbN neuron firing rate as a function of the total amplitude of synchronous inputs. (**e**) The normalized CbN firing rates as a function of the amplitude of the synchronized inputs. (**f**) CbN neuron firing rate as a function of the CV of the inhibitory conductances. (**g–j**) Similar plots to (**c–f**), but for simulations with different-size inputs (dark purple, based on a single distribution, light purple based on nine other different distributions) and uniform inputs (40 × 5 nS, black circles). (**k**) Violin plots showing the simulated CbN neuron firing rate with 100 different distributions of different-size inputs (not syn), and the two largest inputs or the two smallest inputs synchronized. (**l**) As in (**k**) but normalized to the nonsynchronized firing rate. The summary data are shown as the mean ± SEM. Figure 6—source data 1.Dynamic clamp experiments and simulations for syncrony.

Previous dynamic clamp studies based on uniform PC input sizes revealed the potential importance of synchrony in regulating the firing of CbN neurons (Gauck and Jaeger, 2000; Person and Raman, 2012a). We extended this approach to assess the effects of synchrony for different-size PC inputs. We performed dynamic clamp experiments with both uniform-size inputs (Figure 6a) and variable-size inputs (Figure 6b) in each cell. We first looked at the effects of synchrony on the firing rate of CbN neurons. In the absence of synchrony, uniform-size inputs generated a baseline conductance with low variability and CV (Figure 6aii and iii) that resulted in a low baseline firing rate of CbN neurons (25 spikes/s; Figure 6aiv). Synchronizing either 25 or 50% of the uniform inputs (Figure 6ai) increased the variability (Figure 6aii) and the CV (Figure 6aiii) of the total inhibitory conductance, which then resulted in a robust increase in the firing rate of CbN neurons (45 spikes/s with 25% synchrony, 1.8-fold; 88 spikes/s with 50% synchrony, 3.5 fold; Figure 6aiv and v; Figure 6c, black).

For nonuniform inputs (small: 16 × 3 nS; medium: 8 × 12 nS; large: 2 × 30 nS), the generated conductance was more variable (Figure 6bii and iii), resulting in a higher baseline firing rate of CbN neurons (43 spikes/s; Figure 6biv), which is similar to Figure 4h–i. Synchronizing 50% of all inputs (small, medium, and large inputs, Figure 6bi, left) increased the variability (Figure 6bii, left) and CV (Figure 6biii*,* left) of the conductance, which then elevated the firing of CbN neurons from 43 spikes/s to 91 spikes/s (Figure 6biv*,* left; Figure 6c). The firing rates for 50% synchrony with nonuniform and uniform inputs were comparable (Figure 6aiv and biv), but because baseline firing rates were higher for nonuniform inputs, their relative increase in CbN firing was much lower (2.1-fold for nonuniform inputs compared to 3.5-fold for uniform inputs, p<0.0001; Figure 6av and bv). We also examined the effect of synchronizing 25 or 50% of small or medium inputs (Figure 6bi**,** right). Synchronizing 25 or 50% of either small or medium inputs barely increased the variability and CV of the conductance and led to minimal increases in the firing of CbN neurons (Figure 6bii–v**,** right). These observations suggest that the effects of PC synchrony primarily depend on the total amplitude of synchronized inputs (Figure 6d and e). The firing rates of nonsynchronized and synchronized inputs for either uniform or nonuniform input sizes are readily explained by the influence of the CV of the inhibitory conductance on the CbN firing rate (Figure 6f).

Simulations allowed us to explore the effects of a full range of synchrony levels, which would be impractical to test experimentally (Figure 6g–j). We examined uniform inputs (40 × 5 nS) and determined the effect of synchronizing 1, 2, … 20 inputs (Figure 6g–j, black open circles). We also examined nonuniform-size inputs drawn from the full distribution of sizes (Figure 2f, red), with a total conductance of 200 nS, and determined the firing rates evoked when we synchronized different combinations of inputs (Figure 6g–j, dark purple*:* for one distribution of input sizes*;* light purple*:* for nine other distributions of input sizes). The effects of synchrony on firing rates and the dependence on the CV of the conductance were qualitatively similar for simulations (Figure 6g–j) and dynamic clamp experiments (Figure 6c–f). There was a great deal of scatter in the firing rates plotted as a function of the percentage of synchronized inputs (Figure 6g, dark purple), but much less scatter when the firing rates were plotted as a function of the total amplitude of the synchronized inputs (Figure 6h, dark purple). This is consistent with our observation that synchronizing many small- or medium-size inputs does not have much effect on the firing frequency of CbN neurons (Figure 6bi–v*,* right*;* Figure 6c and d). For nonuniform inputs, synchrony has less influence on firing rates because the baseline firing rates are higher (Figure 6e and i). Nonetheless, synchrony of even a small number of large inputs can robustly elevate firing rates. As shown in simulations, synchronizing the two largest inputs can elevate CbN firing rate by approximately 20%, while synchronizing the two smallest inputs barely changed CbN firing rate (Figure 6k and l).

**Figure 7.**
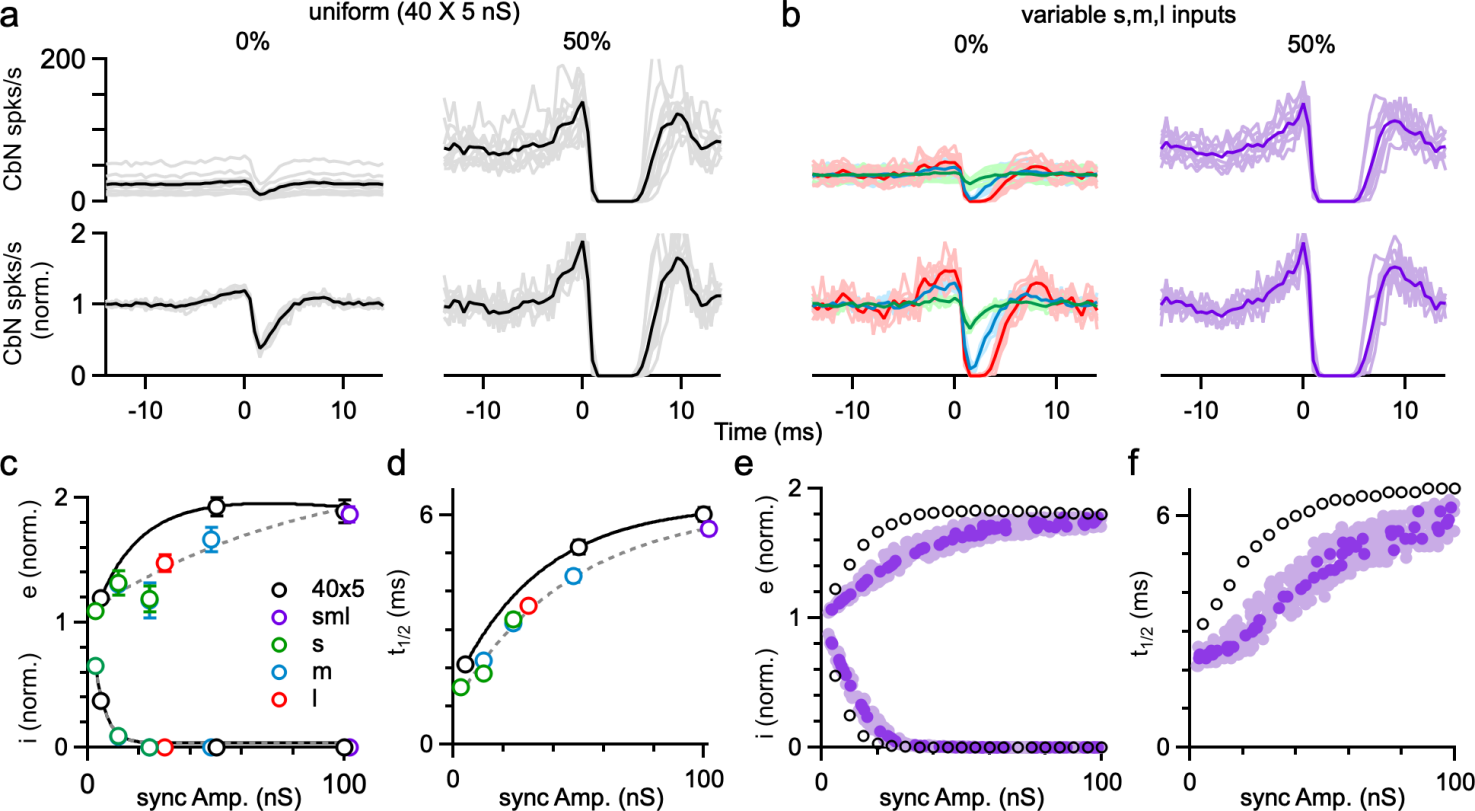
The influence of Purkinje cell (PC) synchrony on the spike timing of cerebellar nuclei (CbN) neurons for uniform- and variable-size PC inputs. (**a**) Top: the cross-correlograms of PC input and CbN neuron spiking for nonsynchronous inputs (left) and 50% synchronous inputs (right) with uniform-size inputs. Individual cells (gray) and averages (black) are shown. Lower: as above, but normalized to the baseline firing rate. (**b**) As in (**a**) but for different-size inputs (as in Figure 6). The cross-correlograms of small (green), medium (blue), and large (red) inputs for unsynchronized inputs (left) and 50% synchronous inputs (right, purple) are shown. (**c**) Summary plots of the excitation (**e**), inhibition (**I**) as a function of the amplitude of synchronized inputs. (**d**) As in (**c**) but for half-decay time (*t*_1/2_). (**e, f**) As in (**c, d**), but for simulations. The summary data are shown as the mean ± SEM. Figure 7—source data 1.Cross-correlograms for synchrony.

We also examined the influence of PC synchrony on the spike timing of CbN neurons in dynamic clamp experiments of Figure 6. Synchrony strongly altered the cross-correlograms of PC inputs and CbN firing, especially for the uniform-size inputs (Figure 7a and b). For uniform inputs (5 nS), single unsynchronized inputs weakly influenced the spike timing of CbN neurons (19% increase; 62% decrease; Figure 7a, left). Also, 50% synchrony of uniform inputs silenced CbN neurons for several milliseconds, and this was proceeded by a prominent increase in CbN spiking (89% increase; Figure 7a, right). This preceding increase in CbN spiking is similar to what was observed for medium and large inputs due to the autocorrelation function of PC firing (Figures 2 and 3) and reflects a disinhibitory period due to the synchronous pauses in PC spikes. This indicates that many small, perfectly synchronized uniform inputs function equivalently as a single large input in controlling the spike time of CbN neurons. For asynchronous nonuniform inputs, as in Figure 2, the influence of individual inputs on the timing of CbN firing depended on the size of the input, and single large inputs could have a very large influence on the timing of CbN spiking (47% increase; 100% decrease; Figure 7b, left, red). Synchronizing 50% of all nonuniform inputs generated a cross-correlogram similar to that of synchronizing 50% uniform inputs (Figure 7a and b, right). The strength of excitation (Figure 7c, top), amplitude of inhibition (Figure 7c, bottom), and duration of inhibition (Figure 7d) were dependent on the synchrony amplitude, as was the case for simulations (Figure 7e and f). For both dynamic clamp experiments and simulations, synchronizing the same amplitude of inputs led to larger timing effects for uniform inputs than for nonuniform inputs (Figure 7c–f), likely because the CbN firing rates with PC synchrony are lower for uniform inputs. These findings establish that PC synchrony and single large inputs are functionally equivalent in controlling the spike timing of CbN neurons, and for nonuniform inputs, the effects of synchrony on CbN spike timing depend on the total size of synchronized inputs.

### Synchronously pausing uniform- or variable-size PC inputs

**Figure 8.**
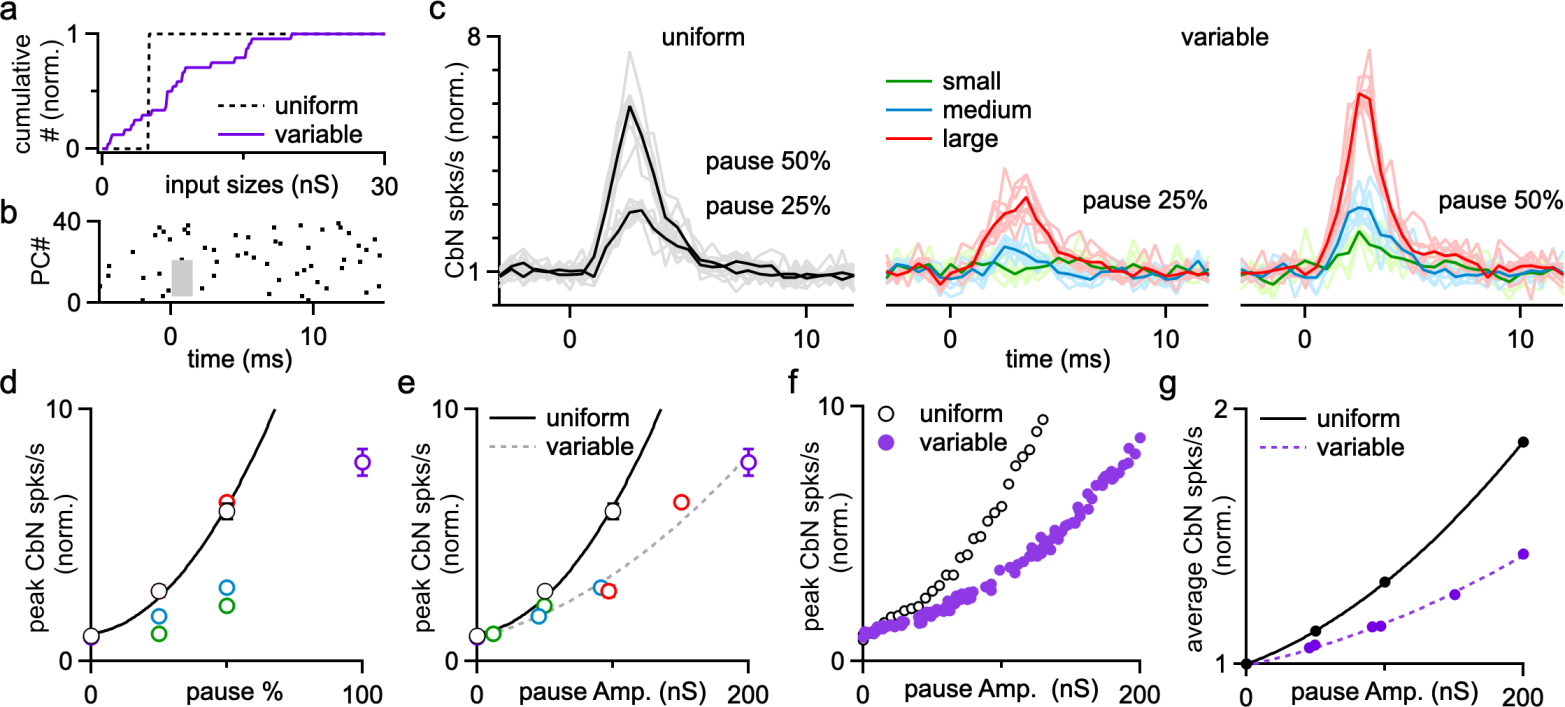
The influence of Purkinje cell (PC) pauses on the firing of cerebellar nuclei (CbN) neurons for uniform- and variable-size PC inputs. Dynamic clamp experiments and simulations were conducted with either uniform or variable inputs in which different percentages of PC inputs were paused for 2 ms. (**a**) Normalized cumulative plots of conductances of PC inputs for a uniform (40 × 5 nS, dashed line) and a variable distribution (purple). (**b**) Raster plot shows example spiking for 40 PC inputs with spikes eliminated in a 2-ms period (gray region) in 50% of the PCs. (**c**) Normalized histograms showing the relative changes in CbN neurons firing for uniform (black) and variable (colored) PC inputs, when PC inputs were paused for 2 ms in 25 and 50% of the inputs. For variable inputs, three different populations of inputs were paused: the smallest, those in the middle of the distribution, and the largest inputs. Average values (bold) and individual cells (faint lines, n = 8) are shown. (**d**) The normalized CbN firing rates as a function of the percentage of paused inputs for uniform (black symbols fit with solid black line) and variable inputs (colored). (**e**) The normalized CbN firing rates as a function of the total amplitude of paused inputs for uniform (black) and variable inputs (colored). Fits are shown for uniform inputs (solid black line) and variable inputs (dashed line). (**f**) As in (**e**), but for simulations where different subsets of PC inputs were paused for 2 ms. (**g**) The normalized overall CbN firing rates as a function of the total amplitude of paused inputs for uniform (black) and variable (purple) inputs from a simulation where different subsets of PC inputs were paused for 2 ms every 50 ms. Fits are shown for uniform inputs (solid black line) and variable inputs (dashed purple line). The summary data are shown as the mean ± SEM. Figure 8—source data 1.Dynamic clamp experiments and simulations for PC pauses.

Brief synchronous pauses of PC firing can also promote CbN neuron firing at rapid time scale (Gauck and Jaeger, 2000; Han et al., 2020). Such pauses in PC firing can arise from inhibition by the synchronous firing of electrically coupled MLIs (Lackey et al., 2023), CF synaptic activation of MLIs (Coddington et al., 2013; Szapiro and Barbour, 2007), and synchronous CF suppression of PC firing through ephaptic signaling (Han et al., 2020). Dynamic clamp studies with uniform-size inputs have shown that pausing a fraction of PC inputs reliably activates CbN neurons (Han et al., 2020). Here we used dynamic clamp studies and simulations to explore the effects of PC pauses on CbN neuron firing with uniform or nonuniform input sizes (Figure 8a). We first performed dynamic clamp experiments in which we paused the firing of 25, 50, and 100% of the PC inputs for 2 ms (Figure 8b). This is illustrated by raster plot showing that inputs from 50% of the PCs were briefly eliminated (Figure 8b, gray box). Pauses in PC firing led to large, short-latency increases in CbN neuron firing for both uniform inputs (Figure 8c; Han et al., 2020) and nonuniform inputs (Figure 8c and d). For nonuniform inputs, we compared the effects of pausing the firing of different-size ranges of PC inputs by pausing the smallest, largest, and middle range of inputs. The larger the size of the paused inputs, the larger the resulting increases in CbN neuron firing (Figure 8c and d), and the magnitude of firing rate increases were determined by the total amplitude of the paused inputs (Figure 8e). Pausing produced larger relative increases in CbN neuron firing for uniform-size PC inputs than that for nonuniform PC inputs (Figure 8c–e), which is likely a secondary consequence of the higher baseline firing frequencies for nonuniform inputs. Simulations where the firing of random combinations of PC inputs was paused for 2 ms produced similar results (Figure 8f). Therefore, brief pauses in PC firing evoke transient increases in CbN neuron firing, and the effects are determined by the total amplitude of paused PC inputs. We also found that CbN firing quickly returned to baseline levels after the transient increase following brief PC pauses (Figure 8c). Consequently, PC pauses only moderately increased the average firing rates of CbN neurons, as shown for a pause interval of 50 ms (Figure 8g). The pause interval did not influence the properties of the transient increases in CbN firing evoked by pauses, but did affect the magnitude of the changes in average firing frequency. For 20 ms, 50 ms, and 100 ms intervals between the 2 ms pauses where all PC spikes were eliminated, the average CbN firing frequency increase was 3.2-, 1.9-, and 1.5-fold for uniform inputs, and 2.1-, 1.5-, and 1.2-fold for nonuniform inputs, respectively. In summary, while the transient increase in CbN firing by PC pauses primarily depends on the total amplitude of paused PC inputs, the overall changes of CbN firing rate at longer time scales depend on both the total amplitude of paused PCs inputs and the pause intervals.

## Discussion

**Figure 9.**
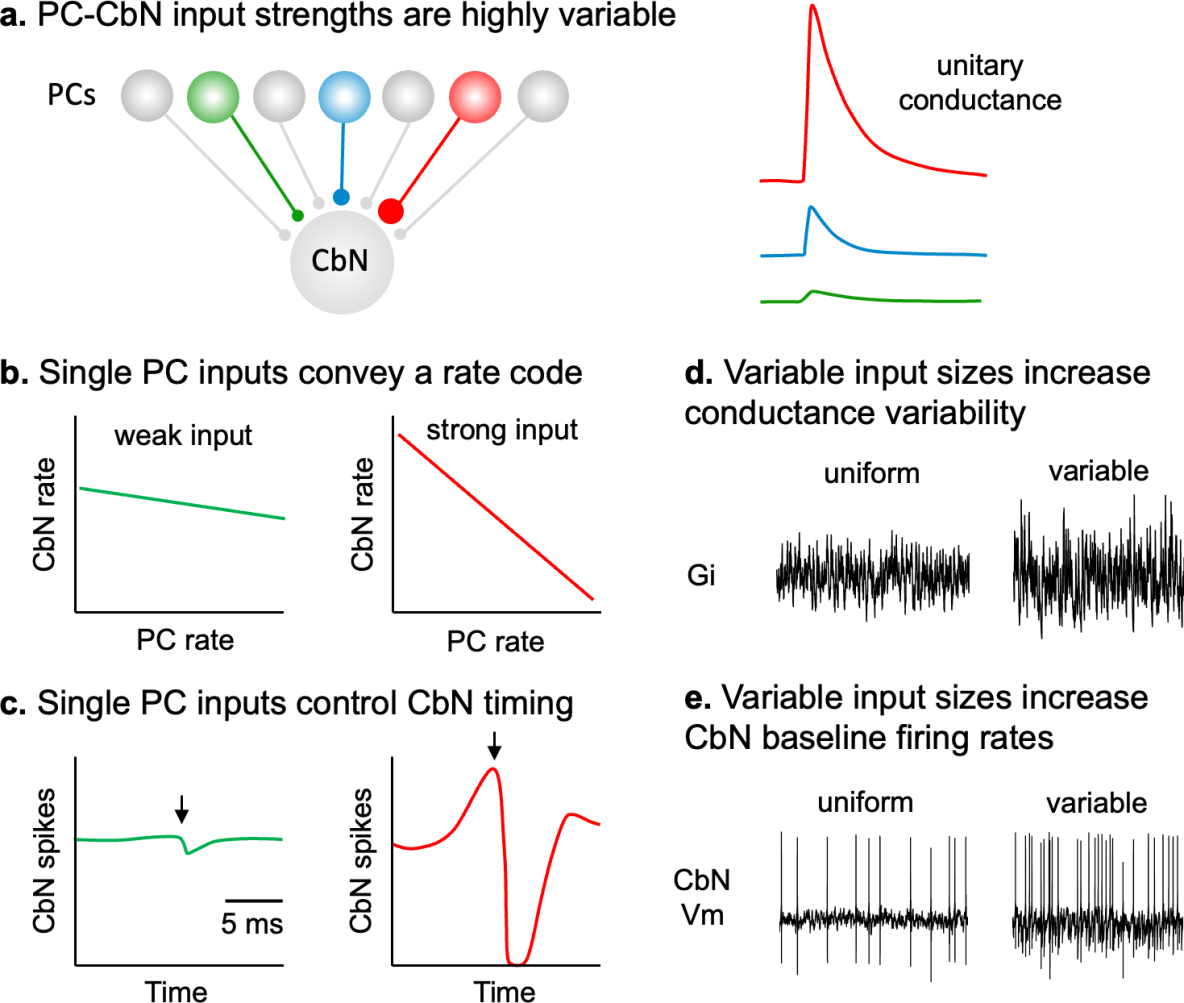
The main consequences of having variable-size Purkinje cell–cerebellar nuclei (PC-CbN) synaptic inputs. (**a**) Left: schematic showing that CbN neurons are innervated by numerous PCs that have very different input strengths. Right: examples of unitary inhibitory conductances from PCs that range in size from very weak (green), medium-sized (blue), to strong (red) (adapted from Figure 1a–c). (**b**) CbN neuron firing rate as a function of input PC firing rate showing linear rate code conveyed by a weak (green) and a strong (red) PC input. (**c**) PC-CbN neuron cross-correlograms show the effects of a weak (green) and a strong (red) PC input. A single strong PC input is highly effective at controlling CbN neuron firing on rapid time scales. As expected, PCs transiently suppress CbN neuron firing, but there is also a preceding increase in CbN firing that arises from the statistics of PC firing and their refractory periods. (**d**) For the same total inhibition, the variability of the total inhibitory conductance is much larger for variable-size inputs than for uniform input sizes. (**e**) Compared to uniform-sized inputs, variable PC input sizes increase the baseline firing rate of CbN neurons as a result of elevated variability in the total inhibitory conductance (**d**). This has important secondary consequences. For variable PC input sizes, elevated basal CbN neuron firing rates reduce the ability of synchronizing or transiently pausing PC firing to promote CbN neuron firing.

Our finding that single PC to CbN inputs are highly variable in size has important implications for how PCs control the firing of CbN glutamatergic projection neurons (Figure 9). We find that individual PCs can strongly influence the firing of CbN neurons, and that PC to CbN synapses are well suited to both implement rate codes and precisely regulate the timing of CbN neuron firing (Figure 9a–c).

### Comparison to previous studies

The approach we took to examine the influence of PCs on CbN neuron firing is similar to that used in previous studies of the influence of PCs on CbN neuron firing (Gauck and Jaeger, 2000; Pedroarena and Schwarz, 2003; Person and Raman, 2012a), but we build upon those studies in several ways. As in earlier studies, we determined the properties of individual PC inputs and confirmed that they had very rapid kinetics (Person and Raman, 2012a). We provide a much more extensive characterization of input sizes (n = 157) than previous studies and show that the distribution of input sizes is skewed, with the largest inputs almost 100 times larger than the smallest inputs (Figure 1). The high Cl- concentration internal we used in our recordings provides superior stability and sensitivity to detect such variability in input size. We then used dynamic clamp to study how PCs control the firing of CbN neurons as in Person and Raman, 2012a, but instead of assuming uniform PC input sizes, we based the PC input sizes on the measured PC-CbN conductances (Figure 2). We first confirmed that many small desynchronized PC inputs are highly effective at suppressing CbN firing, and that synchrony effectively controls the timing and rate of PC firing for small uniform-size PC inputs (Person and Raman, 2012a). We then revealed that the variable PC input sizes have important implications for PC-CbN transmission. A single large input is functionally equivalent to many small, perfectly synchronized inputs. This allows PC inputs to influence the rate and timing of CbN firing as previously proposed (Person and Raman, 2012a), but without requiring a high degree of PC synchrony. We also extended previous studies by using realistic PC firing patterns in our dynamic clamp experiments. This allowed us to determine cross-correlograms for individual inputs and led to the finding that individual inputs have a surprisingly large effect on CbN firing. Previously it was shown that synchronous PC inputs initially suppress CbN neuron firing, which is followed by a *subsequent* increase in firing (Person and Raman, 2012a). Here we found that for spontaneously firing PCs, a period of strong disinhibition *precedes* suppression by a PC input (Figure 3). Lastly, we complemented our dynamic clamp studies with simple simulations that faithfully reproduced our experimental findings, which allowed us to explore the influence of different parameters.

### The variable distribution of PC-CbN input sizes

We showed that there are developmental differences in the distributions of PC-CbN synapse sizes. In older animals (P23–32, Figure 1a–c and e–f, n = 83), the PC input sizes are more variable and there are more large inputs. This developmental transition indicates that PC-CbN synapses are refined during maturation, potentially because of plasticity mechanisms (Aizenman et al., 1998; Broersen et al., 2023; Morishita and Sastry, 1996; Ouardouz and Sastry, 2000). The implications of such variability in the strength of PC-CbN synapses in cerebellar computation can be shown by a recent study describing how the cerebellum encodes vestibular and neck proprioceptive information (Zobeiri and Cullen, 2022). Each PC encodes both vestibular and proprioceptive information, but some CbN neurons exclusively encode body motion and others encode vestibular information (Brooks and Cullen, 2009; Zobeiri and Cullen, 2022). It was proposed that this arises from the linear summation of converging PCs with different weights (Zobeiri and Cullen, 2022). Our observation of the variable distribution of PC-CbN inputs provides evidence for the requisite different weights of PC inputs.

The developmental changes in synaptic strengths we observe are reminiscent of other synapses. For retinal ganglion cell inputs to lateral geniculate nucleus, in young mice, numerous weak inputs innervate each LGN neuron, but in mature mice, this gives way to highly skewed input sizes that include some very strong inputs (Hooks and Chen, 2020; Jiang et al., 2022; Liang and Chen, 2020; Litvina and Chen, 2017). Excitatory inputs in the superior colliculus are refined in a comparable manner (Lu and Constantine-Paton, 2004). For inhibitory inputs to LSO neurons, in P1–5 mice there are many small and no large inputs, but by P9–14 the input sizes are highly skewed, and some inputs are very large (Gjoni et al., 2018; Kim and Kandler, 2003). In the cortex, input sizes are highly variable and are often approximated by a lognormal distribution (Buzsáki and Mizuseki, 2014; Dorkenwald et al., 2022; Melander et al., 2021). Thus, the distribution of PC to CbN input sizes we observe in P23–32 mice is similar in many ways to the distribution of input sizes of other types of synapses. Moreover, network simulations of balanced excitatory and inhibitory circuits have shown that adding variability to the distribution of input sizes disrupts the classical asynchronous irregular state of network activity and leads to rich, nonlinear responses (Brunel, 2000; Wardak and Gong, 2021).

### Nonuniform PC input sizes elevate the basal firing rate of CbN neurons

An important consequence of having highly variable PC-CbN inputs is that it elevates the basal firing rates of CbN neurons (Figures 4, 6,, 9d and e). With the same total inhibition, uniform small PC inputs are particularly effective at suppressing the firing of CbN neurons. Nonuniform-size PC inputs elevate the CV of inhibition, increasing the fluctuations of membrane potential and elevating the firing of CbN neurons. Simulations based on an integrate-and-fire model with a passive cell and a well-defined firing threshold were able to replicate the findings of dynamic clamp experiments. This suggests that CbN neuron firing is effectively driven by the membrane potential fluctuations generated by PC inputs, despite the presence of many excitatory inputs and intrinsic conductances. The high basal firing rate of CbN neurons generated by nonuniform-size inputs helps explain the relatively high firing rates of CbN neurons in vivo amid strong PC inhibition (10–50 Hz) (Eccles et al., 1974; LeDoux et al., 1998; McDevitt et al., 1987; Rowland and Jaeger, 2008; Rowland and Jaeger, 2005; Thach, 1975; Thach, 1970; Thach, 1968). Membrane potential fluctuations have also been shown to be important to spike generation in other cell types (Kuhn et al., 2004; Tiesinga et al., 2000; Yarom and Hounsgaard, 2011).

### Implications of nonuniform input sizes on rate codes

Our findings established that there is an inverse linear relationship between the firing rates of each PC input and the targeted CbN neuron (Figures 5 and 9b; Person and Raman, 2012b; Wu and Raman, 2017). The extent to which the firing rate of an individual PC input regulates the firing rate of a CbN neuron simply depends on the size of the input, and our simulations suggest that single PCs are capable of regulating the firing of a CbN neuron from 35 to 125 spikes/s (Figure 5g; for a 37 nS input varied from 0 to 160 spikes/s). Regardless of the sizes of the input that are varied, the changes in the firing rate of CbN neurons can be readily explained by the alterations in the total inhibitory conductance. It was not obvious prior to these experiments that there would be such a simple relationship between input size, input firing rate, and output firing rate, given that CbN firing rates depend on both the amplitude and CV of inhibition. In practice, fluctuations in the inhibitory conductance are quite large for variable-size inputs, and changing the rate of one input has a relatively small influence on the CV of the inhibition, so the magnitude of the inhibitory conductance dominates the effect. This model allows all different-size PC inputs to reliably convey a simple rate code, with the large PC inputs being particularly effective.

### Implications of nonuniform inputs on temporal codes

Previously it was also thought that an individual PC input has a small influence on the spike timing of a CbN neuron, and that it is necessary to synchronize the firing of many PCs to precisely entrain CbN firing (Heck et al., 2013; Person and Raman, 2012a). Our cross-correlation analysis shows that this is not the case: large inputs (30 nS) eliminated CbN neuron firing for several milliseconds, and even small inputs (3 nS) transiently reduced CbN neuron firing by almost 40% (Figure 2). The sizes of PC inputs, the rapid kinetics of PC-CbN IPSCs (Person and Raman, 2012a), the high firing rate of PCs (Thach, 1968; Zhou et al., 2014), and the strong tendency of CbN neurons to depolarize (Raman et al., 2000) together shape the pattern of cross-correlograms and contribute to precise control of CbN neuron spike timing (Najac and Raman, 2015; Özcan et al., 2020). Thus, PC-CbN synapses are well suited to regulating both the rate and precise timing of CbN firing (Figure 9b and c).

Surprisingly, there was an excitatory response in CbN neuron firing preceding the suppression by PCs in the cross-correlogram of PC inputs and CbN neuron firing (Figure 2). This excitation is a consequence of the autocorrelation function in PC firing (Ostojic et al., 2009), and it will be most prominent for rapidly firing cells (Figure 3). When PCs fire at frequencies lower than 50 spikes/s, the excitatory component is small and lasts for several milliseconds, but when PCs fire at over 100 spikes/s, the excitatory component increases in amplitude and decreases in duration. This indicates the firing rates of PCs will affect the way they control the timing of CbN neuron firing. The observation that PCs in zebrin– regions of the cerebellum tend to fire faster than PCs in zebrin+ regions suggests different timing control of CbN neurons by zebrin+ and zebrin– PCs (Zhou et al., 2014). The effective excitation might also be present for other inhibitory synapses (Arlt and Häusser, 2020; Blot et al., 2016).

The observed distribution of PC to CbN synapse amplitudes, combined with the firing properties of PCs in vivo, allow us to make several predictions about PC-CbN neuron cross-correlograms in vivo. It is expected that PCs to CbN synapses are sufficiently large that prominent suppression should be apparent in cross-correlograms in vivo. The variability of PC-CbN synapses amplitudes suggests that there will be considerable variability in the extent and duration of spike suppression that is comparable to Figure 2. Single large inputs are expected to eliminate CbN neuron firing for several milliseconds, but smaller, weaker connections are expected to be more prevalent. Our findings also predict a disinhibitory component preceding the suppression that will be particularly large for rapidly firing PCs.

### Implications for the influence of synchronous firing and synchronous pauses of PCs

Variable PC input sizes have important implications for how PC synchronous firing and pauses regulate the firing of CbN neurons. First, the relative influence of PC synchrony on the firing rate of CbN neurons is reduced because variable input sizes elevate the basal firing rates of CbN neurons (Figures 4, 6,, 9d and e). Second, the effect of PC synchrony depends upon which inputs are synchronized. Synchronizing 50% of the small inputs (corresponding to 31% of the total inputs) increased CbN firing by just 13%, whereas synchronizing the two largest PC inputs to a CbN neuron increased the CbN firing rate by 21% (Figure 6k and l). These findings suggest that a high degree of synchrony is not prerequisite for an appreciable influence. Therefore, studies that fail to detect a high degree of synchrony in PC simple spike firing in vivo (Herzfeld et al., 2023b) do not exclude the physiological relevance of PC synchrony in regulating CbN neurons. Similarly, we show that the relative influence of PC pauses is also reduced with variable-size inputs as a secondary effect of the high baseline firing rate, and is also determined by the total amplitude of paused inputs. Thus, the influence of PC inputs on their CbN targets depends upon the amplitudes of the different inputs, the firing patterns of the PCs, and the degree to which they are synchronized or paused.

### Overall summary

Our initial findings that PC to CbN synapses are highly variable in size (Figure 9a), and that some are extremely large, have important implications for how PCs regulated the firing of CbN neurons and the output of the cerebellum. We found that individual large PC inputs strongly suppress the average firing rates of CbN neurons (Figure 9b) and also transiently silence them for several milliseconds (Figure 9c), thereby allowing them to convey both rate information and precise temporal information. An important characteristic of the strong temporal control of CbN neuron firing by individual PC inputs and the statistics of PC firing is that in PC-CbN cross-correlograms, inhibition is preceded by an apparent increase in CbN firing that looks like excitation. We also found that nonuniform input sizes elevate baseline firing rates by increasing the variability of the inhibitory conductance (Figure 9d and e). This has the secondary effect of reducing the efficacy of synchronous firing and synchronous pauses in elevating CbN firing.

## Methods

**Key resources table:** 

Reagent type (species) or resource	Designation	Source or reference	Identifiers	Additional information
Strain, strain background (*Mus musculus*)	C57BL/6	Charles River Laboratories	N/A	
Chemical compound, drug	NBQX disodium salt	Abcam	Ab120046	
Chemical compound, drug	(R,S)-CPP	Abcam	Ab120160	
Chemical compound, drug	Strychnine hydrochloride	Abcam	Ab120416	
Chemical compound, drug	SR95531 (gabazine)	Abcam	Ab120042	
Chemical compound, drug	CGP 55845 hydrochloride	Abcam	Ab120337	
Chemical compound, drug	QX-314 chloride	Abcam	Ab120118	
Software, algorithm	Igor Pro	WaveMetrics (https://www.wavemetrics.com/order/order_igordownloads6.htm)	RRID:SCR_000325	Version 6.37

### Animals

C57BL/6 wild-type mice (Charles River Laboratories) of both sexes aged P10–P32 were used for acute slice experiments (for dynamic clamp experiments, P26–P32). All animal procedures were conducted in accordance with the National Institutes of Health and Animal Care and Use Committee guidelines and protocols approved by the Harvard Medical Area Standing Committee on Animals (animal protocol #1493).

### Slice preparation

Mice were anesthetized with ketamine/xylazine/acepromazine and transcardially perfused with warm choline ACSF solution (34°C) containing in mM 110 choline Cl, 2.5 KCl, 1.25 NaH_2_PO4, 25 NaHCO_3_, 25 glucose, 0.5 CaCl_2_, 7 MgCl_2_, 3.1 Na pyruvate, 11.6 Na ascorbate, 0.002 (R,S)-CPP, 0.005 NBQX, oxygenated with 95% O_2_/5% CO_2_. To prepare sagittal CbN slices, the hindbrain was removed, a cut was made down the midline of the cerebellum and brainstem, and the halves of the cerebellum were glued down to the slicing chamber. Sagittal slices (170 μm) were cut using a Leica 1200S vibratome in warm choline ACSF (34°C). Slices were transferred to a holding chamber with warm ACSF solution (34°C) containing in mM 127 NaCl, 2.5 KCl, 1.25 NaH_2_PO_4_, 25 NaHCO_3_, 25 glucose, 1.5 CaCl_2_, 1 MgCl_2_, and were recovered at 34°C for 10 min before being moved to room temperature for another 20–30 min until recordings begin.

### Electrophysiology

Whole-cell voltage/current-clamp recordings were performed on large neurons (>70 pF) in the lateral and interposed DN. These large cells are primarily glutamatergic projection neurons (Baumel et al., 2009; Turecek et al., 2016; Uusisaari et al., 2007).

For voltage-clamp recordings of unitary PCs inputs to CbN neurons, Borosilicate glass electrodes (1–2 MΩ) were filled with a high-chloride (E_Cl_ = 0 mV) internal containing in mM 110 CsCl, 10 HEPES, 10 TEA-Cl, 1 MgCl_2_, 4 CaCl_2_, 5 EGTA, 20 Cs-BAPTA, 2 QX314, and 0.2 D600, adjusted to pH 7.3 with CsOH. BAPTA was included to prevent long-term plasticity (Ouardouz and Sastry, 2000; Pugh and Raman, 2006; Zhang and Linden, 2006). The osmolarity of internal solution was adjusted to 290–300 mOsm. Series resistance was compensated up to 80%, and the calculated liquid junction potentials were around 5 mV and were left unsubtracted. CbN neurons were held at –30 to –40 mV. All experiments were performed at 34–35°C in the presence of 5 μM NBQX to block AMPARs, 2.5 μM (R,S)-CPP to block NMDARs, 1 μM strychnine to block glycine receptors, and 1 μM CGP 55845 to block GABA_B_Rs, with a flow rate of 3–5 ml/min. A glass monopolar stimulus electrode (2–3  MΩ) filled with ACSF was placed in the white matter between the CbN and the cerebellar cortex to activate PC axons. Minimal stimulation was used to determine the amplitudes of single PC-CbN inputs. The stimulus intensity was adjusted so that synaptic inputs were activated in approximately half the trials in a stochastic manner. The sample size was achieved by performing as many recordings as possible within a limited period of time.

For dynamic clamp experiments, Borosilicate glass electrodes (3–4 MΩ) were filled with an internal containing (in mM) 145 K-gluconate, 3 KCl, 5 HEPES, 5 HEPES-K, 0.5 EGTA, 3 Mg-ATP, 0.5 Na-GTP, 5 phosphocreatine-tris2, and 5 phosphocreatine-Na2, adjusted to pH 7.2 with KOH. The osmolarity of internal solution was adjusted to 290–300 mOsm. Series resistances were less than 15 MΩ and were not compensated. Voltages were corrected for a liquid junction potential of 10 mV. Cells were held at –65 to –75 mV between trials. All experiments were performed at 34–35°C in the presence of 5 μM NBQX, 2.5 μM (R,S)-CPP, 5 μM SR 95531 (Gabazine), and 1 μM strychnine to block most synaptic transmission.

### Dynamic clamp experiments

The total inhibitory conductance (200 nS) from all converging PCs in each CbN neuron was based on previous estimation of 40 PCs with a size of 5 nS (after depression) (Person and Raman, 2012a). Uniform-size inputs are studied in Figures 3, 4c–g,–6. For the uniform inputs with different firing frequencies used in Figure 3, the number of the inputs was adjusted so that the total inhibitory conductances were the same for all groups (after depression, 12 × 20 nS at 49 Hz, 9 × 20 nS at 83 Hz, 6 × 20 nS at 122 Hz, and 9 × 20 nS for Poisson inputs). For the uniform inputs with different sizes used in Figure 4c–g, the input sizes varied from 2.5 nS to 40 nS (after depression), and the number of inputs was varied to maintain a total inhibitory conductance of 200 nS. Forty PC inputs with a size of 5 nS were used in Figure 6.

Dynamic clamp experiments with different-size inputs were performed in Figures 2a–e–4h–i—6,–8. To generate different-size inputs reflecting the distribution of the unitary PC-CbN input conductances measured in P23–32 animals, the amplitudes of the unitary conductances were corrected for depression (× 0.4) and the effects of high Cl- internal (scale down by a factor of 2.3) (Bormann et al., 1987; Gjoni et al., 2018; Sakmann et al., 1983). In experiments where the effects on average firing frequency were determined (Figure 4h and i) and in Figure 8, input sizes were randomly drawn from the experimentally determined distributions of input sizes (Figure 2f, red) until the total inhibitory conductance reached 200 nS. In experiments where the spike-triggered averages were to be determined, we used a simplified distribution. We estimated the simplified distribution by computing the ratio and weighted averages for different ranges of input sizes. In Figures 2a–e–5, we approximated the distribution with small (16 × 3 nS), medium (10 × 10 nS), and large (2 × 30 nS) inputs. Approximating the small inputs with 16 inputs of the same size made it possible to determine the spike-triggered average with a much better signal to noise than if the inputs were different sizes. We took a similar approach in Figure 6, but we adjusted the number and size of medium-size inputs to make it easier to assess the effects of 50 and 25% synchrony (16 × 3 nS small, 8 × 12 nS medium, and 2 × 30 nS large inputs).

We based the timing of PC firing (Figure 2—figure supplement 1) on in vivo recordings from our previous study (Han et al., 2020). The average firing frequency ranges from 61 spikes/s to 180 spikes/s (Figure 2—figure supplement 1a and b). The distribution of ISIs of firing in the 10 PCs was well approximated with lognormal functions (Figure 2—figure supplement 1c), and the relationship between the mean and the standard deviation (sd) of lognormal distributions fits was well approximated with a linear function: sd = –0.00154 + 0.583 * mean (Figure 2—figure supplement 1d). Therefore, we used this linear function to calculate the σ of a desired firing frequency (1/mean) and generated an artificial ISI distribution of PC firing based on lognormal function with the designated mean and sd. The ISI distributions of PC firing used in Figures 2 and 6 were artificial lognormal distributions with a firing frequency of 83 Hz (Figure 2—figure supplement 1c) and 80 Hz (Figure 2—figure supplement 1e and f), respectively. The three ISI distributions of PC firing with different firing frequencies used in Figure 3 are from in vivo recordings (Figure 2—figure supplement 1a and b), and the Poisson distribution without a refractory period was generated with an exponential function aiming at a desired frequency (80 Hz). The ISI distribution of PC firing used in Figure 4 is from in vivo recordings with a firing frequency of 100 Hz (Figure 2—figure supplement 1a and b). The ISI distributions of PC firing with different firing frequencies used in Figure 5 are artificial lognormal distributions generated from the μ and σ of desired frequencies. The approach is shown for artificial lognormal distributions of 40, 80, 120, and 160 spikes/s (Figure 2—figure supplement 1e and f). Individual PC spike trains were generated by randomly drawing ISIs from the designated ISI distribution, and spike trains from each PC were combined as a final spike train with the inputs from all PCs. This spike train was then convolved with a unitary PC input with a rise time of 0.1 ms and a decay time of 2.5 ms (Khan et al., 2022). The reversal potential for inhibitory conductances was set at –75 mV (after correcting for junction potential).

Excitatory conductances were based on the AMPA component of mossy fiber (MF) excitatory postsynaptic currents (EPSCs) characterized in previous studies (Wu and Raman, 2017), with a rise time of 0.28 ms, a decay time of 1.06 ms, and an amplitude of 0.4 nS (reflecting depression). They estimated that 20–600 MFs converged on each CbN neuron, with unknown firing frequencies, so the excitatory conductances were relatively unconstrained. We adjusted the frequency of MF EPSCs so that the basal firing rate of CbN neurons was maintained at 20–40 Hz in the presence of the inhibitory conductance. The average baseline excitatory conductance was 20–30 nS.

To avoid a drastic increase in spike frequency and the following adaptation of CbN neurons resulting from big changes in the variability of conductances, we ramped up the CV of the inhibitory conductances in the beginning of each trial so that the firing rate of CbN increased gradually from the hyperpolarization state. Each conductance was repeated in the same neuron for 3–4 trials as technical replicates. For synchrony experiments, PC inputs were synchronized 100% in their spike times. Therefore, synchronizing 10 5 nS inputs is equivalent to having one big input with a size of 50 nS.

### Analysis

Recordings were obtained using Multiclamp 700B (Molecular Devices), sampled at 50 kHz and filtered at 4 kHz, and collected in Igor Pro (WaveMetrics). Dynamic clamp recordings were performed with an ITC-18 computer interface controlled by mafPC in Igor Pro (WaveMetrics). Data were analyzed using custom-written scripts in MATLAB (MathWorks) and Igor Pro (WaveMetrics). Autocorrelation and cross-correlation analyses were performed by generating accumulative histograms of spikes distribution within a 20 ms time window centering all spikes from the reference file (self-reference for autocorrelation, and PCs spike times for cross-correlation), and normalized by the total spikes number of the reference file and the bin size of the histograms (i.e., the Δ*t*). All summary data are shown as the mean ± SEM unless otherwise indicated. The distributions of unitary PC input sizes in young and juvenile animals in Figure 1f were compared with a Kolmogorov–Smirnov test. The unpaired *t*-test was performed with Welch’s correction.

### Simulations

Simulations were performed (Figures 2f–h–4j—8f, g) to complement dynamic clamp experiments. A point-conductance, single-compartment model was generated to model the CbN neuron and its synaptic inputs. The membrane potential (*V*) of a CbN neuron with a membrane capacitance ($C_{m}$) follows the equation

$$C_{m}\frac{dV}{dt}=g_{E}\left(t\right)\left(V_{E}-V\right)+g_{I}\left(t\right)\left(V_{I}-V\right)+g_{L}\left(V_{L}-V\right)$$

where $V_{E}=0$ mV and $V_{I}\;=\;-75$ mV are the excitatory and inhibitory reversal potentials, and $g_{E}\left(t\right)$ and $g_{I}\left(t\right)$ are time-dependent excitatory and inhibitory conductances. The leak was modeled by a constant leak conductance $g_{L}$ with a reversal potential $V_{L}$ . A spike occurs when the membrane potential V of the model neuron reaches the threshold θ, at which point there is a refractory period of 2 ms during which the model neuron remains inactive, and the neuron is then reset to $V_{r}$ . $g_{E}\left(t\right)$ and $g_{I}\left(t\right)$ were generated as described for dynamic clamp experiments. Simulations were performed using the BRIAN simulation environment in Python. The simulation code is available on GitHub at https://github.com/asemptote/PC-DCN-different-size-inputs (Wardak, 2023).

In Figure 2f–h, simulations were performed for 10 cells with input distributions drawn randomly from the corrected empirical input sizes (Figure 2f, red) such that the total conductance was 200 nS. Each cell was run for 160,000 s and cross-correlograms were computed for each input. The parameters used were $C_{m}=50$ pF, 20,000 excitatory events per second, θ=-50 mV, $V_{r}=-60$ mV, $g_{L}=8.8$ nS, and $V_{L}=-40$ mV. Inhibitory spike trains were randomly chosen by drawing ISIs from the empirically fitted lognormal distribution such that the mean rate was 83 Hz. These were repeated for input distributions drawn from a rescaled version of the input sizes with scaling factors of 0.5 (Figure 2—figure supplement 3a) and 1.5 (Figure 2—figure supplement 3b).

In Figure 4j, simulations were performed for 100 cells with input distributions drawn randomly from the corrected empirical input sizes, along with 200 cells with varying numbers of uniform-size inputs such that the total conductance was 200 nS. Each cell was run for 10 s, and the firing rates and inhibitory conductance CV were recorded. The generated inhibitory conductance waves were saved for use in experiments in Figure 4i. The parameters used were $C_{m}=200$ pF, 23,650 excitatory events per second, θ=-50 mV, $V_{r}=-60$ mV, $g_{L}=5$ nS, and $V_{L}=-10$ mV. For the cells with varying input sizes, inhibitory spike trains were randomly chosen by drawing ISIs from the empirically fitted lognormal distribution such that the mean rate was 80 Hz, while for the cells with uniform-size inputs, the ISIs were drawn from an in vivo recording with a firing frequency of 100 Hz (Figure 2—figure supplement 1).

In Figure 5, simulations were performed for 10 cells with input distributions drawn randomly from the corrected empirical input sizes such that the total conductance was 200 nS. For each input to each cell, 16 simulations were run for 100 s where the rate of a given input was varied between 0 and 160 Hz while keeping the other inputs at a fixed rate (80 Hz). The average firing rate and mean inhibitory conductance were recorded. These were then repeated with input scaling factors of 0.5 (Figure 5—figure supplement 1A) and 1.5 (Figure 5—figure supplement 1B). Parameters used were as in Figure 4. Inhibitory spike trains were randomly chosen by drawing ISIs from the empirically fitted lognormal distribution such that the mean rate was 80 Hz.

In Figures 6g–j and 7e and f, simulations were performed for 10 cells with input distributions drawn randomly from the corrected empirical input sizes such that the total conductance was 200 nS. For each cell, 100 simulations were run for 1600s where a random subset of the input sizes was synchronized, and the firing rate (Figure 6g–j) and cross-correlation statistics (Figure 7e and f) were obtained as in Figure 2. Forty additional simulations were run in this manner for a cell with 40 uniform-size inputs, synchronizing a different number of inputs in each simulation. Parameters used were as in Figure 5.

In Figure 8 and g, simulations were performed with an input distribution drawn randomly from the corrected empirical input sizes such that the total conductance was 200 nS. Each cell was run for 16,000 s where spiking was eliminated from one of 100 random subsets of inputs for 2 ms in each 20 ms time period. The normalized peak CbN firing rate was determined by the averaged increase in firing relative to the baseline defined as the average rate over the 5 ms periods before the pause. The peak time used for each cell was determined by the histogram corresponding to 100% of the inputs paused. The parameters used were $C_{m}=70$ pF, 23,650 excitatory events per second, θ=-50 mV, $V_{r}=-60$ mV, $g_{L}=20$ nS, and $V_{L}=-49.9$ mV. A refractory period of 1 ms was used. Inhibitory spike trains were randomly chosen by drawing ISIs from the empirically fitted lognormal distribution such that the mean rate was 80 Hz. These simulations were repeated for a cell with uniform-size inputs where the parameters were kept the same apart from an excitatory rate of 25,000 events per second.

### Code availability

The simulation code is available on GitHub at https://github.com/asemptote/PC-DCN-different-size-inputs.

## Data Availability

All data used in this study are originally generated, with the exception of some of the PC-CbN unitary input sizes in Figure 1d (Turecek et al., 2017) and Figure 1e (Khan and Regehr, 2020), and PC spike times in Figure 2 - figure supplement 1 (Han et al., 2020). The data for the new recordings of PC-CbN unitary inputs in Figure 1 and the data for dynamic clamp studies and simulations in Figure 2 to Figure 8 can be found in the source data for the respective figure. Code availability: The simulation code is available on GitHub at https://github.com/asemptote/PC-DCN-different-size-inputs (copy archived at Wardak, 2023).

## Additional files

MDAR checklist
