# Supplementary material for: Implications of variable synaptic weights for rate and temporal coding of cerebellar outputs
Source: eLife. 2024 Jan 19;13:e89095. doi: 10.7554/eLife.89095 (PMC10798666; doi:10.7554/eLife.89095)
Supplement: Peer review history

# Editor report: Editor's evaluation

**DOI:** 10.7554/eLife.89095.sa0

**Authors:** Sacha B Nelson

**Affiliations:** https://ror.org/05abbep66 Brandeis University United States

This important manuscript emphasizes some previously ignored aspects of synaptic communication between Purkinje neurons and their targets in the cerebellar nuclei. The evidence supporting the main conclusions is solid.

# Decision letter

**DOI:** 10.7554/eLife.89095.sa1

**Affiliations:** https://ror.org/05abbep66 Brandeis University United States

Our editorial process produces two outputs: i) public reviews designed to be posted alongside the preprint for the benefit of readers; ii) feedback on the manuscript for the authors, including requests for revisions, shown below. We also include an acceptance summary that explains what the editors found interesting or important about the work.

**Decision letter after peer review:**

Thank you for submitting your article "Implications of variable synaptic weights for rate and temporal coding of cerebellar outputs" for consideration by *eLife*. Your article has been reviewed by 2 peer reviewers, and the evaluation has been overseen by Sacha Nelson as the Reviewing and Senior Editor.

The reviewers have discussed their reviews with one another, and the Reviewing Editor has drafted this to help you prepare a revised submission.

Essential revisions:

As you can see from the comments below, most of the concerns raised by reviewers can be addressed through textual changes. Both reviewers felt that the scholarship of the manuscript could be improved and that additional quantitative summary and statistical testing was warranted (for example the degree of increase of skewness).

There was less clear cut agreement over the conductance measurements. The concern was raised that the high chloride used distorts this measurement in ways that were not very convincingly controlled or justified. Possible solutions to this issue raised included:

1) Redoing a subset of the measurements with more physiological chloride

2) Providing a more rigorous analysis of the overestimate of the conductance (i.e. a more detailed justification of the correction factor used.

*Reviewer #1 (Recommendations for the authors):*

I detail some of the main problems with this study below. Most of these issues are fixable.

1. Scholarship issues. Many of the claims about background knowledge in the paper lack citations, and many of these "claims" are strawmen. A few, but not exhaustive examples:

a. "Previously it was thought that PC-CbN synapses are uniformly small and that single PC inputs have little influence on the firing of CbN neurons." This statement is made repeatedly in the abstract, introduction, and results, yet no citation is provided but is essential. Undermining the point of the authors, the Person and Raman 2012 study shows a skewed distribution of unitary synaptic strengths of PCs. It also shows that a 10 nS input, equivalent to one input, is sufficient to suppress firing of a nuclear cell consistently (Figure S2 in that study). Person and Raman 2012 b discuss the potential anatomical underpinnings of skewed synaptic strengths. Here is the relevant text in 2012b: "Perhaps more importantly, as the authors state explicitly, their "apparently excessive" numerical estimate for average convergence is based on the simplifying assumption that each Purkinje cell ramifies to the same extent on all nuclear cells. They point out, however, that Purkinje neurons consistently make non-uniform contacts onto nuclear cells, occasionally "erupting" into "numerous (~50) boutons all in contact with the same cell body." Ramón y Cajal similarly observed that each Purkinje neuron axon formed six to eight "nests" onto as many cells (Chan-Palay, 1977). Such dense terminal perisomatic plexes are also mentioned in other descriptions of Purkinje axonal arbors (Chan-Palay, 1977; Bishop et al., 1979; De Zeeuw et al., 1994; Wylie et al., 1994; Teune et al., 1998; Sugihara et al., 2009), suggesting that they are a common specialization of Purkinje neuron terminals. Palkovits et al. (1977) propose that each Purkinje neuron may have 3-6 primary targets, while providing weak input to many more, and conversely that nuclear cells may only receive strong somatic input from "several"-that is, a relatively small number of- Purkinje cells (see also Sugihara et al., 2009)."

b. The either-or nature of the synchrony vs rate coding hypotheses is not backed up by a citation "Leading theories" which ones?

c. "Individual PCs are thought to have limited influence on CbN firing" this is another statement that is not backed up by any literature I am familiar with. Indeed Person and Raman show in a supplemental figure that the equivalent of single inputs effectively inhibit CbN cells.

d. Paragraph 2 Introduction: "a rate code does not apply" – this is sloppy language that does not appropriately frame the state of the field. There are many possible explanations for deviations from inverse firing rate relationships, one of which is synchrony, but others include diverse excitatory afferents to the nuclei and cortex, neuromodulatory influence or other mechanisms.

2. Some of the main experimental claims are either not novel, mischaracterize previous literature, or lack rigor themselves:

A. "The distribution of input sizes we observed in P10-20 animals was similar to previous studies (P13-29, n=30) (Person and Raman, 2012a), and the skewed distribution of input sizes was only apparent when many individual inputs were characterized in P23-32 mice." This conclusion is unconvincing. There is skew in both the young and the old populations, and if it's more skewed in the old is it more skewed than the overlapping age population shown before. I find this language borderline disingenuous. Moreover the cited study shows a skewed distribution of synaptic strengths.

B. The estimate of the synaptic conductances is problematic. The use of high Cl^-^ interferes with the measurement, then they engage in some hand-waving arguments to come to an unconstrained estimate of 3-30 nS. This is not well constrained and will introduce unnecessary confusion into the literature.

C. There is too little statistical and quantitative information in the main text. For example, the first full paragraph of page 4 states: "All inputs had effects on the timing of CbN neuron firing, but larger inputs had much bigger effects. As shown in the cross-correlograms of input timing and CbN neuron spiking, small inputs produced a small transient decrease in CbN neuron spiking, while medium sized inputs strongly reduced CbN neuron spiking, and large inputs transiently shut down CbN neurons for approximately 2 ms. No average or statistics are provided in the subsequent text either. This style makes it difficult to grasp the magnitude of any of these differences, and in any event, it seems obvious that graded inhibition would produce graded effect sizes.

D. The text states that the refractory period is correlated with the amount of CbN firing and refers to Figure 3. I see no evidence that a correlation was computed from this figure, nor are the statistics of any correlation reported in the text. That said, the potential relationship between CbN firing and PC refractoriness is interesting, but poorly explained in the text. A clearer mechanistic insight would be valuable, beyond "This excitation is a consequence of the autocorrelation function in PC firing", which does not shed much light on the matter. It might be helpful to show the raster plots of spike-triggered inputs to better decipher relationships here. (Rasters for Figure 2D would also be valuable).

E. For the data in Figure 5, what accounts for the very high firing rates of CbN neurons (200 spikes/s)? The plots in Figure 5 could be more intuitive with some work.

*Reviewer #1 (Recommendations for the authors - second review):*

The authors have thoroughly addressed my concerns. The revision beter frames the findings, both in terms of novelty and significance than the original submission. It will be of value to the field.

*Reviewer #2 (Recommendations for the authors):*

Main concerns for the authors:

The manuscript is very dense and constitute an important dataset for the field. The use of dynamic clamp is very interesting and enhances experimental options. I have however important concerns that I think could help clarify the manuscript:

– My first concern is the choice of the distribution with a different number of small, medium and large input size. I understand that it is necessary to keep the total conductance constant, but it raises a doubt on the relevance of the data in Figures 2 and 4c for example. Indeed, a few large inputs could be considered as many small inputs synchronized. Similarly, few large inputs obviously lead to a high CV by construction, while many small inputs reduce the CV. What is really tested in all these different protocols is a bit confusing.

– How is played this distribution on PC inputs? In episodes or continuously?

– A set of in vivo PC discharge is used, but where is it coming from?

– The correction made to approximate conductance is somehow arbitrary. I would tone down the assumption that this is the first demonstration that PC-DCN inputs are large and variable. Indeed, in preceding studies, authors already suggested that PC-DCN inputs are quite large in the recordings, albeit with different recording conditions.

In Person and Raman, Nature 2012 "The high unitary amplitude suggests that individual Purkinje-to-nuclear contacts are quite strong", referring to an article that should be cited here as well:

Pedroarena, C. M. and Schwarz, C. Efficacy and short-term plasticity at GABAergic synapses between Purkinje and cerebellar nuclei neurons. J. Neurophysiol. 89, 704-715 (2003).

Actually the interplay between rate coding and temporal coding has been also addressed in:

Özcan, O. O., Wang, X., Binda, F., Dorgans, K., De Zeeuw, C. I., Gao, Z., Aertsen, A., Kumar, A., and Isope, P. (2020). Differential Coding Strategies in Glutamatergic and GABAergic Neurons in the Medial Cerebellar Nucleus. The Journal of Neuroscience, 40(1), 159-170.

– I am not a specialist in modeling, but I would imagine that since CbN neurons have active conductances (Ih, Cav3 channels), it is surprising that a simple integrate and fire reproduce fully CbN spiking behavior. The choice of model could be more discussed. In the MS, the discussion around rebound firing is also very limited.

– The concept of the refractory period accounting for the excitatory effect preceding PC inputs is not well explained, and the simple hypothesis that spikes were elicited at the time of the lowest inhibitory conductance might be enough.

– Figure 4 The frequency observed in CbN neurons seems much higher than in any other study. Similarly, what is the actual firing rate of neurons recorded in Figure 5 without inhibitory inputs?

[Editors' note: further revisions were suggested prior to acceptance, as described below.]

Thank you for resubmitting your work entitled "Implications of variable synaptic weights for rate and temporal coding of cerebellar outputs" for further consideration by *eLife*. Your revised article has been evaluated by Sacha Nelson (Senior Editor) and a Reviewing Editor.

The manuscript has been improved but there are some remaining issues that need to be addressed, as outlined below:

Some additional modification of the text is warranted to address the remaining concern of Reviewer #2. In addition, a third reviewer was consulted who raised some of the same concerns raised earlier about statistical analyses of the underlying distribution and the validity of the extrapolation to measurements made in symmetrical chloride. Perhaps some of the points raised to address these issues in the previous reply to reviewers could be included in the manuscript to shore up these points.

*Reviewer #2 (Recommendations for the authors):*

The authors have definitely improved their manuscript, results are better explained and discussed. Based on my previous review, I would have two remaining requests:

– Related to Fig2g and my question about rebound firing, the authors answered

"With regard to the issue of rebound firing, under our experimental conditions, we see no indication of rebound firing". In Fig2g, it seems that a rebound firing occurs for large inputs. It could be important to mention it as synchronization (e.g. by climbing fiber inputs) would also give the same outcome.

– Related to the discussion about input size vs synchrony, uniform vs nonuniform inputs, I think that a summary figure would help catch the take-home message.

*Reviewer #3 (Recommendations for the authors):*

In Wu et al., the authors describe how inputs of variable synaptic weights influence rate and temporal output coding in neurons of the cerebellar nuclei. This is valuable research that advances our understanding of this important question within the field of cerebellar encoding, with experiments that give solid (or mostly solid) evidence for their claims.

In this manuscript, the authors focus on an important question in the cerebellar field: how do high-firing inhibitory Purkinje cells influence the output of neurons in the cerebellar nuclei (CbN) which are themselves spontaneously active? They use classical techniques: whole-cell electrophysiology combined with extracellular stimulation and dynamic-clamp experiments. They identify that there is a wide range of input strengths made onto CbN neurons from Purkinje cells and explore what this diversity of input strength might mean for CbN neuron output. Using dynamic clamp, they parametrically alter input synchrony, comparing how CbN output is altered when they alter inputs of different sizes. Some of their findings are counterintuitive, as they show that right before a strong inhibition of CbN firing caused by Purkinje cell activation, there is a transient enhancement of firing which is due to Purkinje cell refractory period. Other findings are more straight-forward – for e.g. they show that large inputs can strongly modulate firing and thus there is an inverse rate code. They look at synchrony and show that the impact on CbN firing depends on input variability, something that has not previously been addressed in detail. These findings are interesting and add to our understanding of how Purkinje cell synapses may influence CbN output.

1. One of the main claims of the paper is that they uncover that there is variable Purkinje cell input to CbN neurons. However, this finding is based on recordings with symmetrical Cl^-^ internal that is not physiological. They use a calculation based on the literature (based on other synapses that may have different channel properties) to adjust their measurements to physiological values, but the appropriateness of this adjustment is not evident. This makes me question how solid this finding is, as there is no experimental validation. In fact, it is not entirely clear how much their finding differs from previous findings – they say that they show a different distribution, but they do not verify it is different statistically. I think they need to either strengthen their experimental evidence for this finding (which I acknowledge would be a challenging thing to do) or weaken their claims.

2. The authors talk about Purkinje cell autocorrelation as being the cause of the modulation of firing before the Purkinje cell spike reduces firing in CbN neurons. I find this section hard to follow, and given the general readership of *eLife*, I think the authors could do a better job explaining this in a more accessible way.

# Reply: Author response

**DOI:** 10.7554/eLife.89095.sa2

Essential revisions:

Reviewer #1 (Recommendations for the authors):

I detail some of the main problems with this study below. Most of these issues are fixable.

1. Scholarship issues. Many of the claims about background knowledge in the paper lack citations, and many of these "claims" are strawmen. A few, but not exhaustive examples:

a. "Previously it was thought that PC-CbN synapses are uniformly small and that single PC inputs have little influence on the firing of CbN neurons." This statement is made repeatedly in the abstract, introduction, and results, yet no citation is provided but is essential. Undermining the point of the authors, the Person and Raman 2012 study shows a skewed distribution of unitary synaptic strengths of PCs. It also shows that a 10 nS input, equivalent to one input, is sufficient to suppress firing of a nuclear cell consistently (Figure S2 in that study). Person and Raman 2012 b discuss the potential anatomical underpinnings of skewed synaptic strengths. Here is the relevant text in 2012b: "Perhaps more importantly, as the authors state explicitly, their "apparently excessive" numerical estimate for average convergence is based on the simplifying assumption that each Purkinje cell ramifies to the same extent on all nuclear cells. They point out, however, that Purkinje neurons consistently make non-uniform contacts onto nuclear cells, occasionally "erupting" into "numerous (~50) boutons all in contact with the same cell body." Ramón y Cajal similarly observed that each Purkinje neuron axon formed six to eight "nests" onto as many cells (Chan-Palay, 1977). Such dense terminal perisomatic plexes are also mentioned in other descriptions of Purkinje axonal arbors (Chan-Palay, 1977; Bishop et al., 1979; De Zeeuw et al., 1994; Wylie et al., 1994; Teune et al., 1998; Sugihara et al., 2009), suggesting that they are a common specialization of Purkinje neuron terminals. Palkovits et al. (1977) propose that each Purkinje neuron may have 3-6 primary targets, while providing weak input to many more, and conversely that nuclear cells may only receive strong somatic input from "several"-that is, a relatively small number of- Purkinje cells (see also Sugihara et al., 2009)."

We thank the reviewer for the comment and have rephrased our statements to be more precise. Person and Raman made a simplifying assumption when they used 40 uniform size PC inputs in their dynamic clamp studies (Person and Raman 2012a). This was a reasonable strategy, based on the distribution of input sizes they observed (Person and Raman 2012a), which is less skewed compared to what we observed when studying a larger number of inputs at different developmental stages (see response to Review #1, point 2 below). In Person and Raman 2012b, there was a nice discussion of the anatomical studies suggesting heterogenous PC boutons on CbN neurons, but it was difficult to relate such anatomical studies to PC input strength. Here we want to point out that the heterogeneity of PC-CbN connections and its implications on PC-CbN transmission have not been addressed experimentally, and previous studies using dynamic clamp were based on 40 uniform size inputs (Han et al., 2020; Person and Raman, 2012; Wu and Raman, 2017). All of these studies (including our own) made the same simplifying assumption and ignored the issue of different size inputs.

McDevitt et al., 1987 is often cited to address whether individual PC can regulate CbN neuron firing, as by Person and Raman 2012b: “In decerebrate cats, for example, spontaneous activity of such Purkinje-nuclear pairs is not correlated, and firing rate modulation in response to periodic sensory stimuli is not consistently reciprocal, leading to the conclusion that single Purkinje afferents are insufficient to regulate the spiking behavior of nuclear cell targets (McDevitt et al., 1987).” In our study, we find that this conclusion is not necessarily true since large PC inputs powerfully influence the firing of CbN neurons, which supports the importance of studying the consequences of different input sizes. We predicted similar observations to be made in PC-CbN neuron cross-correlograms in vivo.

The reviewer also referred to Figure S2 of Person and Raman 2012a, in which they studied how the time-course of single IPSC inputs influences the firing activity of CbN neurons, in the absence of other inhibitory or excitatory inputs. That experiment was not designed to show the strength of single PC input, and more appropriate experiments (e.g. testing the inhibition by single PC input in the presence of many other inhibitory and excitatory inputs) were not performed. Furthermore, although there were dynamic clamp experiments in which 40 asynchronous PC inputs drove CbN neuron firing, the effects of individual inputs were not analyzed (Person and Raman 2012a). Instead, the paper emphasized the effects of synchrony: “With partial synchrony, nuclear neurons time-lock their spikes to the synchronous subpopulation of inputs, even when only 2 out of 40 afferents synchronize” (Person and Raman 2012a). Therefore, we do not think the effects of individual PC inputs have been characterized in previous studies.

b. The either-or nature of the synchrony vs rate coding hypotheses is not backed up by a citation "Leading theories" which ones?

We fully agree with the reviewer that rate and time codes are not mutually exclusive. We have modified the manuscript to clarify this point. However, there is controversy in the field, and we think it is appropriate to point this out. Below are a few examples:

“After all, years of research have shown that much of the information sent to motor areas from the cerebellum is conveyed by a rate code — a modulation in the firing frequency of neurons in which the precise timing of the spikes is irrelevant because the rate is averaged over tens to hundreds of milliseconds… So, at least in theory, this would allow inputs from different groups of Purkinje cells to become synchronized for brief periods of time, and, when a high degree of temporal precision is required (for instance, at the beginning or end of a movement), to control the exact timing of spikes in nuclear neurons. To control other aspects of movement, such as amplitude or speed, the cerebellum might switch Purkinje cells back to the asynchronous mode and modulate their firing frequency up or down, thus regulating the firing rate of nuclear neurons.” (Medina and Khodakhah, 2012).

“Through an ingenious set of experiments, Person and Raman describe one such computational principle: how a (nuclear) neuron decodes the temporal structure of its synchronous (Purkinje cell) inhibitory inputs while ignoring the asynchronous ones… But a confounding limitation of such temporal decoding is that the probability of firing entrained spikes is reduced so much that nuclear neurons cannot ratecode — that is, the persistent activity of the asynchronous inputs reduces the probability of a nuclear neuron firing entrained spikes to such an extent that it can no longer encode the firing rate of its synchronous inputs in its own firing rate. This is problematic, because it has been known for more than 40 years that the activity rates of individual nuclear and Purkinje neurons correlate with movement and thereby with each other.” (Medina and Khodakhah, 2012).

“Information transmission in the brain can occur via changes in firing rate or via the precise timing of spikes. Simultaneous recordings from pairs of Purkinje cells in the floccular complex reveals that information transmission out of the cerebellar cortex relies almost exclusively on changes in firing rates rather than millisecond-scale coordination of spike timing across the Purkinje cell population.” (Herzfeld et al., 2023)

Such controversy can also be found in many other studies, as concluded in the introduction (Abbasi et al., 2017; Brown and Raman, 2018; Cao et al., 2012; Gauck and Jaeger, 2000; Heck et al., 2013; Herzfeld et al., 2023a; Hoehne et al., 2020; Hong et al., 2016; Medina and Khodakhah, 2012; Payne et al., 2019; Person and Raman, 2012a, 2012b; Sedaghat-Nejad et al., 2022; Stahl et al., 2022; Sudhakar et al., 2015; Walter and Khodakhah, 2009; Wu and Raman, 2017).

Alternatively, Person and Raman 2012a have promoted a view that is compatible with a combination of a rate code and a synchrony code, or more generally that timing and rate can both matter.

We have tried to provide a more nuanced discussion of the issues of synchrony, timing and rate to address the reviewer’s concerns.

c. "Individual PCs are thought to have limited influence on CbN firing" this is another statement that is not backed up by any literature I am familiar with. Indeed Person and Raman show in a supplemental figure that the equivalent of single inputs effectively inhibit CbN cells.

Please refer to our response to point a above.

d. Paragraph 2 Introduction: "a rate code does not apply" – this is sloppy language that does not appropriately frame the state of the field. There are many possible explanations for deviations from inverse firing rate relationships, one of which is synchrony, but others include diverse excitatory afferents to the nuclei and cortex, neuromodulatory influence or other mechanisms.

We have revised the text to clarify this point.

2. Some of the main experimental claims are either not novel, mischaracterize previous literature, or lack rigor themselves:

We do not agree with this general statement. We have responded to the specific issues raised by the reviewer below.

A. "The distribution of input sizes we observed in P10-20 animals was similar to previous studies (P13-29, n=30) (Person and Raman, 2012a), and the skewed distribution of input sizes was only apparent when many individual inputs were characterized in P23-32 mice." This conclusion is unconvincing. There is skew in both the young and the old populations, and if it's more skewed in the old is it more skewed than the overlapping age population shown before. I find this language borderline disingenuous. Moreover the cited study shows a skewed distribution of synaptic strengths.

The distribution we observed in P23-32 mice is very different from that shown in a previous study (Person and Raman, 2012a). We therefore feel that our description of the results was fair and reasonable, and it was not “disingenuous.”

Although we have not changed the text regarding this issue, we are open to specific suggestions, provided it is consistent with the differences in the skewness of the distributions in Response Figure 1. The authors also provided a figure (Response Figure 1, that is not reproduced here) in which distributions of input sizes were directly compared for (a) 30 inputs from p13-29 mice (Person and Raman, 2012a, Fig. 1b lower), and (b) 83 inputs from p23-32 mice from this paper (Fig. 1e, corrected for high chloride internal). This direct comparison made the differences in the skew of the distributions obvious.

B. The estimate of the synaptic conductances is problematic. The use of high Cl^-^ interferes with the measurement, then they engage in some hand-waving arguments to come to an unconstrained estimate of 3-30 nS. This is not well constrained and will introduce unnecessary confusion into the literature.

The reviewer’s concern provides us with another chance to address how we constrain the input sizes based on several factors. We used a high Cl^-^ internal to determine the distribution of input sizes because it provided superior stability, better access resistance and higher sensitivity compared to using a low Cl^-^ internal. We scaled down the conductance amplitudes by 2.3, guided by previous findings that the measured chloride conductances were 2-3 times larger for high chloride vs. physiological chloride internals (Bormann et al., 1987; Gjoni et al., 2018; Sakmann et al., 1983). Modest errors in the correction from high to physiological chloride concentrations will not affect our major conclusions but might have small quantitative effects on the influence of single inputs. We performed additional simulations to address this issue (Figure 2 —figure supplement 3; Figure 5 —figure supplement 1).

We also took into account the depression that occurs during high frequency activation of PC inputs (Turecek et al., 2017, 2016; Telgkamp and Raman, 2002; Pedroarena and Schwarz, 2003, reduced to 40% of the initial response), as in previous dynamic clamp studies (Han et al., 2020; Person and Raman, 2012; Wu and Raman, 2017). Based on the corrected distribution of input sizes, we took two major approaches: (1) For dynamic clamp studies we used a simplified distribution based on the experimentally determined distribution of input sizes (16 X 3 nS, 10 X 10 nS, and 2 X 30 nS). We have provided additional clarification in the revised paper, which we hope has addressed the issue. (2) We performed simulations in which we used different size inputs drawn from the experimentally determined distribution. Overall, these approaches were based on experimental data and reasonable assumptions.

C. There is too little statistical and quantitative information in the main text. For example, the first full paragraph of page 4 states: "All inputs had effects on the timing of CbN neuron firing, but larger inputs had much bigger effects. As shown in the cross-correlograms of input timing and CbN neuron spiking, small inputs produced a small transient decrease in CbN neuron spiking, while medium sized inputs strongly reduced CbN neuron spiking, and large inputs transiently shut down CbN neurons for approximately 2 ms. No average or statistics are provided in the subsequent text either. This style makes it difficult to grasp the magnitude of any of these differences, and in any event, it seems obvious that graded inhibition would produce graded effect sizes.

We thank the reviewer for the suggestion and have added more quantification. If there are any additional quantification or statistical tests you would like us to perform, please point them out and we will update the manuscript accordingly.

D. The text states that the refractory period is correlated with the amount of CbN firing and refers to Figure 3. I see no evidence that a correlation was computed from this figure, nor are the statistics of any correlation reported in the text. That said, the potential relationship between CbN firing and PC refractoriness is interesting, but poorly explained in the text. A clearer mechanistic insight would be valuable, beyond "This excitation is a consequence of the autocorrelation function in PC firing", which does not shed much light on the matter. It might be helpful to show the raster plots of spike-triggered inputs to better decipher relationships here. (Rasters for Figure 2D would also be valuable).

We thank the reviewer for their comment and we have provided additional quantification. We appreciate the reviewer’s interest on the issue and suggestions to improve the clarity of the manuscript. In short, the firing statistics of PCs determine their refractory period, which in turn result in different extents of excitation (e.g., amplitude, duration) prior to the inhibition. We have added more explanations in our text. We also think having the raster plots is a good idea and we have added this to the figure (Figure 3 —figure supplement 1). I hope the additional explanation and figures have clarified this point.

E. For the data in Figure 5, what accounts for the very high firing rates of CbN neurons (200 spikes/s)? The plots in Figure 5 could be more intuitive with some work.

We agree with the reviewer that the previous Figure 5 was not very intuitive. We moved most of Figure 5 to a supplemental Figure, provided additional explanation, and retained the parts that were more intuitive. We hope this addresses the reviewer’s concerns.

Reviewer #1 (Recommendations for the authors - second review): The authors have thoroughly addressed my concerns. The revision beter frames the findings, both in terms of novelty and significance than the original submission. It will be of value to the field.

We are pleased that we were able to address all of the reviewer’s concerns.

Reviewer #2 (Recommendations for the authors):

Main concerns for the authors:

The manuscript is very dense and constitute an important dataset for the field. The use of dynamic clamp is very interesting and enhances experimental options. I have however important concerns that I think could help clarify the manuscript:

– My first concern is the choice of the distribution with a different number of small, medium and large input size. I understand that it is necessary to keep the total conductance constant, but it raises a doubt on the relevance of the data in Figures 2 and 4c for example. Indeed, a few large inputs could be considered as many small inputs synchronized. Similarly, few large inputs obviously lead to a high CV by construction, while many small inputs reduce the CV. What is really tested in all these different protocols is a bit confusing.

We understand the reviewer’s concern about our experimental design of the input sizes. Our priority purposes are to reproduce the physiological relevant sizes, ratio, and total inhibitory conductance. As we addressed above in our response to reviewer 1, we based our estimation on the corrected distribution and compute the ratio and weighted averages for different ranges of input sizes. The number of small, medium, and large inputs were chosen to represent their ratio from our recordings. The total inhibitory conductance (200 nS) was chosen based on previous measurement of the maximal evoked inhibitory conductance of CbN neuron in slices (Telgkamp and Raman, 2002; Person and Raman, 2012). We agree that different size inputs inevitably alter the CV, and we systematically showed how the CV of the inhibitory conductance affects CbN neuron firing (Figure 4).

– How is played this distribution on PC inputs? In episodes or continuously?

The different conductance waves were played in episodes, separated with intervals and alternated in sequence between different trials to minimize the effects of adaptation of CbN neuron firing. We have expanded the Methods section to provide additional details about the experiments.

– A set of in vivo PC discharge is used, but where is it coming from?

We thank the reviewer for point this out.

The firing pattern of 10 PCs recorded in vivo used in this study were from our previous studies (Han et al., 2020). In Figure 2 —figure supplement 1, we showed that the ISI distribution of these 10 PCs can be nicely described with lognormal distributions, and we used these artificial PC ISI distributions in most experiments.

– The correction made to approximate conductance is somehow arbitrary. I would tone down the assumption that this is the first demonstration that PC-DCN inputs are large and variable. Indeed, in preceding studies, authors already suggested that PC-DCN inputs are quite large in the recordings, albeit with different recording conditions.

In Person and Raman, Nature 2012 "The high unitary amplitude suggests that individual Purkinje-to-nuclear contacts are quite strong", referring to an article that should be cited here as well:

Pedroarena, C. M. and Schwarz, C. Efficacy and short-term plasticity at GABAergic synapses between Purkinje and cerebellar nuclei neurons. J. Neurophysiol. 89, 704-715 (2003).

Actually the interplay between rate coding and temporal coding has been also addressed in:

Özcan, O. O., Wang, X., Binda, F., Dorgans, K., De Zeeuw, C. I., Gao, Z., Aertsen, A., Kumar, A., and Isope, P. (2020). Differential Coding Strategies in Glutamatergic and GABAergic Neurons in the Medial Cerebellar Nucleus. The Journal of Neuroscience, 40(1), 159-170.

We thank the reviewer for the suggestion and revise our manuscript accordingly. We explained our correction in the response to reviewer 1 (see 2-B).

Please note that previous papers suggest that PC inputs were quite large, but they were still much smaller that the large inputs we observed (over 80 nS). With regard to input sizes, Pedroarena and Schwarz found an average input size of 11.7 nS prior to depression (P12-21 rats at 25-29°C; with high Cl^-^ internal; after correction for high Cl^-^ internal corresponds to an average input size of 3.8 nS prior to depression). Person and Raman found an average input size of 9.4 nS (P13-29 mice at 36°C, with physiological Cl^-^ concentration internal), which is much smaller than the very large inputs we see (see Figure 1 and the response to reviewer 1). Özcan at al. is an interesting and relevant paper and we have cited it in the revised paper.

– I am not a specialist in modeling, but I would imagine that since CbN neurons have active conductances (Ih, Cav3 channels), it is surprising that a simple integrate and fire reproduce fully CbN spiking behavior. The choice of model could be more discussed. In the MS, the discussion around rebound firing is also very limited.

We agree that it is surprising that our simple model is in such good agreement with our dynamic clamp experiments. We would have explored more complex and realistic models had the integrate and fire model failed to recapitulate our experimental findings (p. 4). We think this indicates that our conclusions are not specific to the properties of the CbN neurons and have added to the discussion of this point. With regard to the issue of rebound firing, under our experimental conditions we see no indication of rebound firing.

– The concept of the refractory period accounting for the excitatory effect preceding PC inputs is not well explained, and the simple hypothesis that spikes were elicited at the time of the lowest inhibitory conductance might be enough.

This is an important finding, and we agree with this concern by both reviewers that this part of the manuscript needs clarification. We have revised the figure and provided addition discussion of this point. We hope this issue has been addressed, but we welcome suggestions for ways to clarify this point.

– Figure 4 The frequency observed in CbN neurons seems much higher than in any other study. Similarly, what is the actual firing rate of neurons recorded in Figure 5 without inhibitory inputs?

The high firing frequency of CbN neurons in these experiments likely resulted from the drastic changes in the inhibitory conductance, since we matched the excitatory conductances in the beginning of these experiments to reach a basal CbN firing rate of 30-50 spikes/s. Some of the scenarios we tested in these experiments are extreme cases (e.g., half of the PC inputs pause their firing) and therefore showed very high firing rate of CbN neurons (e.g., 200 spikes/s). The range of the firing rate of CbN neurons have also been observed in other dynamic clamp studies (Wu and Raman, 2017). The firing rate of CbN neurons in our other experiments mostly fall in physiological ranges (less than 100 spikes/s).

[Editors’ note: what follows is the authors’ response to the second round of review.]

The manuscript has been improved but there are some remaining issues that need to be addressed, as outlined below:

Some additional modification of the text is warranted to address the remaining concern of Reviewer #2. In addition, a third reviewer was consulted who raised some of the same concerns raised earlier about statistical analyses of the underlying distribution and the validity of the extrapolation to measurements made in symmetrical chloride. Perhaps some of the points raised to address these issues in the previous reply to reviewers could be included in the manuscript to shore up these points.

Reviewer #2 (Recommendations for the authors):

The authors have definitely improved their manuscript, results are better explained and discussed. Based on my previous review, I would have two remaining requests:

– Related to Fig2g and my question about rebound firing, the authors answered

"With regard to the issue of rebound firing, under our experimental conditions, we see no indication of rebound firing". In Fig2g, it seems that a rebound firing occurs for large inputs. It could be important to mention it as synchronization (e.g. by climbing fiber inputs) would also give the same outcome.

The reviewer refers to an apparent rebound component in CbN firing for large PC inputs (Figure 2g). This might also arise from the autocorrelation function of the PC firing: if there is a PC spike at me t=0, then that PC will not fire for several milliseconds before or after that spike as a result of the refractory period, which then lead to disinhibition and transiently promote firing. This is clear in Figure 3d where the cross-correlogram generated by a Poisson input does not show the disinhibition either before and after the inhibition. We have provided an additional explanation to clarify this point (p. 5).

The issue of climbing fibers and different size inputs is interesting. We focused on simple spikes, and we have not examined the issue of climbing fibers and different size inputs either experimentally or with simulations. Hopefully we will be able to address this issue in great depth in future studies.

– Related to the discussion about input size vs synchrony, uniform vs nonuniform inputs, I think that a summary figure would help catch the take-home message.

This is an excellent suggestion. We have added a new figure to summarize the main take home messages (Figure 9).

Reviewer #3 (Recommendations for the authors):

In Wu et al., the authors describe how inputs of variable synaptic weights influence rate and temporal output coding in neurons of the cerebellar nuclei. This is valuable research that advances our understanding of this important question within the field of cerebellar encoding, with experiments that give solid (or mostly solid) evidence for their claims.

In this manuscript, the authors focus on an important question in the cerebellar field: how do high-firing inhibitory Purkinje cells influence the output of neurons in the cerebellar nuclei (CbN) which are themselves spontaneously active? They use classical techniques: whole-cell electrophysiology combined with extracellular stimulation and dynamic-clamp experiments. They identify that there is a wide range of input strengths made onto CbN neurons from Purkinje cells and explore what this diversity of input strength might mean for CbN neuron output. Using dynamic clamp, they parametrically alter input synchrony, comparing how CbN output is altered when they alter inputs of different sizes. Some of their findings are counterintuitive, as they show that right before a strong inhibition of CbN firing caused by Purkinje cell activation, there is a transient enhancement of firing which is due to Purkinje cell refractory period. Other findings are more straight-forward – for e.g. they show that large inputs can strongly modulate firing and thus there is an inverse rate code. They look at synchrony and show that the impact on CbN firing depends on input variability, something that has not previously been addressed in detail. These findings are interesting and add to our understanding of how Purkinje cell synapses may influence CbN output.

1. One of the main claims of the paper is that they uncover that there is variable Purkinje cell input to CbN neurons. However, this finding is based on recordings with symmetrical Cl^-^ internal that is not physiological. They use a calculation based on the literature (based on other synapses that may have different channel properties) to adjust their measurements to physiological values, but the appropriateness of this adjustment is not evident. This makes me question how solid this finding is, as there is no experimental validation. In fact, it is not entirely clear how much their finding differs from previous findings – they say that they show a different distribution, but they do not verify it is different statistically. I think they need to either strengthen their experimental evidence for this finding (which I acknowledge would be a challenging thing to do) or weaken their claims.

The new reviewer shares some of the concerns with the initial reviewers, and we addressed the issue in response to the other reviewers (we assume that the new reviewer saw the revised paper but did not see our previous response).

Our response to the original reviews: The distribution we observed in P2332 mice is very different from that shown in a previous study (Person and Raman, 2012a). We therefore feel that our description of the results was fair and reasonable, and it was not “disingenuous.”

Although we have not changed the text regarding this issue, we are open to specific suggestions, provided it is consistent with the differences in the skewness of the distributions in Figure 1.

The reviewer’s concern provides us with another chance to address how we constrain the input sizes based on several factors. We used a high Cl^-^ internal to determine the distribution of input sizes because it provided superior stability, better access resistance and higher sensitivity compared to using a low Cl^-^ internal. We scaled down the conductance amplitudes by 2.3, guided by previous findings that the measured chloride conductances were 2-3 times larger for high chloride vs. physiological chloride internals (Bormann et al., 1987; Gjoni et al., 2018; Sakmann et al., 1983). Modest errors in the correction from high to physiological chloride concentrations will not affect our major conclusions but might have small quantitative effects on the influence of single inputs. We performed additional simulations to address this issue (Figure 2 —figure supplement 3; Figure 5 —figure supplement 1).

We also took into account the depression that occurs during high frequency activation of PC inputs (Turecek et al., 2017, 2016; Telgkamp and Raman, 2002; Pedroarena and Schwarz, 2003, reduced to 40% of the initial response), as in previous dynamic clamp studies (Han et al., 2020; Person and Raman, 2012; Wu and Raman, 2017). Based on the corrected distribution of input sizes, we took two major approaches: (1) For dynamic clamp studies we used a simplified distribution based on the experimentally determined distribution of input sizes (16 X 3 nS, 10 X 10 nS, and 2 X 30 nS). We have provided additional clarification in the revised paper, which we hope has addressed the issue. (2) We performed simulations in which we used different size inputs drawn from the experimentally determined distribution. Overall, these approaches were based on experimental data and reasonable assumptions.

Changes we made to respond to this issue in this latest revision:

We mentioned in the discussion that “This distribution is clearly different from that of Person and Raman (2012a)”. Although we think this is fair, we could not make this a statistically robust comparison without having access to the original data in that paper. We therefore think it is appropriate to simply remove this sentence from the discussion. Here we want to emphasize that based on our data, the PC inputs should be treated as a population of nonuniform size inputs, as opposed to the simplifying assumption of uniform inputs that was made in many previous studies.

2. The authors talk about Purkinje cell autocorrelation as being the cause of the modulation of firing before the Purkinje cell spike reduces firing in CbN neurons. I find this section hard to follow, and given the general readership of eLife, I think the authors could do a better job explaining this in a more accessible way.

We appreciate this comment. We very much want people to understand this important point, and we have therefore provided additional clarification (p. 5).
